# TFEB-mediated lysosomal biogenesis and lysosomal drug sequestration confer resistance to MEK inhibition in pancreatic cancer

**DOI:** 10.1038/s41420-020-0246-7

**Published:** 2020-03-11

**Authors:** Ben Zhao, Laura Dierichs, Jiang-Ning Gu, Marija Trajkovic-Arsic, Ralf Axel Hilger, Konstantinos Savvatakis, Silvia Vega-Rubin-de-Celis, Sven-Thorsten Liffers, Samuel Peña-Llopis, Diana Behrens, Stephan Hahn, Jens T. Siveke, Smiths S. Lueong

**Affiliations:** 1 Division of Solid Tumor Translational Oncology, German Cancer Consortium (DKTK, partner site Essen) and German Cancer Research Center, DKFZ, Heidelberg, Germany, Division of Solid Tumor Translational Oncology, German Cancer Consortium (DKTK, partner site Essen) and German Cancer Research Center, DKFZ, Heidelberg, Germany, Essen, Germany; 2 Institute for Developmental Cancer Therapeutics, West German Cancer Center, University Hospital Essen, Essen, Germany, Heidelberg, Germany; 3 Department of Hepatobiliary Surgery, the First Affiliated Hospital of Dalian Medical University, Dalian, Liaoning Province China; 4 Dept Med Oncol, West German Cancer Center, University Hospital Essen, Essen, Germany; 5 Institute for Cell Biology, University Hospital Essen, Essen, Germany; 6 Translational Genomics in Solid Tumors, West German Cancer Center, University Hospital Essen, Essen, Germany; 7 EPO – Experimental Pharmacology and Oncology GmbH Berlin-Buch, Berlin, Germany; 8 Department of Molecular GI-Oncology, Rurh University Bochum, Bochum, Germany

**Keywords:** Targeted therapies, Targeted therapies

## Abstract

Oncogenic *KRAS* mutations are encountered in more than 90% of pancreatic ductal adenocarcinomas. MEK inhibition has failed to procure any clinical benefits in mutant RAS-driven cancers including pancreatic ductal adenocarcinoma (PDAC). To identify potential resistance mechanisms underlying MEK inhibitor (MEKi) resistance in PDAC, we investigated lysosomal drug accumulation in PDAC models both in vitro and in vivo. Mouse PDAC models and human PDAC cell lines as well as human PDAC xenografts treated with the MEK inhibitor trametinib or refametinib led to an enhanced expression of lysosomal markers and enrichment of lysosomal gene sets. A time-dependent, increase in lysosomal content was observed upon MEK inhibition. Strikingly, there was a strong activation of lysosomal biogenesis in cell lines of the classical compared to the basal-like molecular subtype. Increase in lysosomal content was associated with nuclear translocation of the Transcription Factor EB (*TFEB*) and upregulation of *TFEB* target genes. siRNA-mediated depletion of *TFEB* led to a decreased lysosomal biogenesis upon MEK inhibition and potentiated sensitivity. Using LC-MS, we show accumulation of MEKi in the lysosomes of treated cells. Therefore, MEK inhibition triggers lysosomal biogenesis and subsequent drug sequestration. Combined targeting of MEK and lysosomal function may improve sensitivity to MEK inhibition in PDAC.

## Introduction

Pancreatic ductal adenocarcinoma (PDAC) is among the most aggressive and lethal malignant human diseases, with a 5-year survival rate of less than 8%^1^. Given the high frequency of tumors diagnosed at an advanced stage and the high rate of intrinsic and acquired resistance to virtually any therapy, blocking resistance mechanisms remains a key challenge. Recent evidence points to the existence of at least two major molecular subtypes: the quasi-mesenchymal or basal-like and the classical subtypes, with differences in signaling activities and therapy response^2,3^.

Activating mutations in *KRAS* is the most frequent genetic alteration observed in more than 90% of PDAC tumors^4^. These mutations promote proliferation and inhibit apoptosis via the RAF/MEK/ERK and PI3K/AKT pathway^5–7^, thereby making Ras inhibition an attractive drug target. Unfortunately, there are currently no effective therapies for about 30% of all *Ras* mutant human malignancies^8^. This is in part due to lack of target specificity and feedback loops, which have rendered direct *KRAS* targeting very challenging and efforts have been focused on targeting downstream signaling pathways such as MEK/ERK^7,9^. Despite development of highly-specific MEK inhibitors and good on-target efficacy, MEK inhibition has unfortunately failed to show any clinical benefit^10,11^ in PDAC as well as in other cancer entities^12,13^. The mechanisms underlying the inefficacy of MEK inhibition in PDAC are still not well understood. However, combinatorial strategies may constitute promising avenues, as recent combination of MEK and SHP2 inhibition have shown to overcome RTK-mediated pathway reactivation in *KRAS*-mutant tumors^14,15^.

Hydrophobic anticancer agents with weak base properties are easily trapped in the acidic medium of the lysosomes by cation trapping^16^. Such organelle-mediated drug sequestration keeps drugs far from their molecular targets, thereby inducing drug resistance^17^. Additionally, the number of drug-containing lysosomes per cell has been shown to be directly correlated with the extent of resistance^17^ and lysosomal drug accumulation is known to trigger cytoplasmic to nuclear translocation of the transcription factor EB (*TFEB*). *TFEB* is a master regulator of lysosomal biogenesis and autophagy, dependent on the mechanistic target of rapamycin complex 1 (mTORC1)^17,18^. Lysosomal drug sequestration also induces lysosomal exocytosis and hence drug excretion from within target cells^16^. Investigating the role of lysosomal drug sequestration upon MEK inhibition in PDAC is therefore warranted. This is further strengthened by recent reports, where MEK inhibition is reported to elicit protective autophagy in Ras-driven cancer^19^.

meanwhile pathway reactivation and reliance on interferon-mediated signaling has been reported^20,21^, the role of lysosomal drug sequestration of MEK inhibitors in PDAC has not been addressed. We herein report on lysosomal sequestration of MEK inhibitors by PDAC cells in vitro and in vivo and a strong activation of lysosomal biogenesis upon MEKi treatment. Furthermore, we show that disruption of lysosomal biogenesis by TFEB knockdown partially sensitizes PDAC cells to trametinib treatment and demonstrate the presence of trametinib in lysosomes.

## Results

### MEKi treatment triggers expression of lysosome-associated genes in mouse PDAC models

**Fig. 1:**
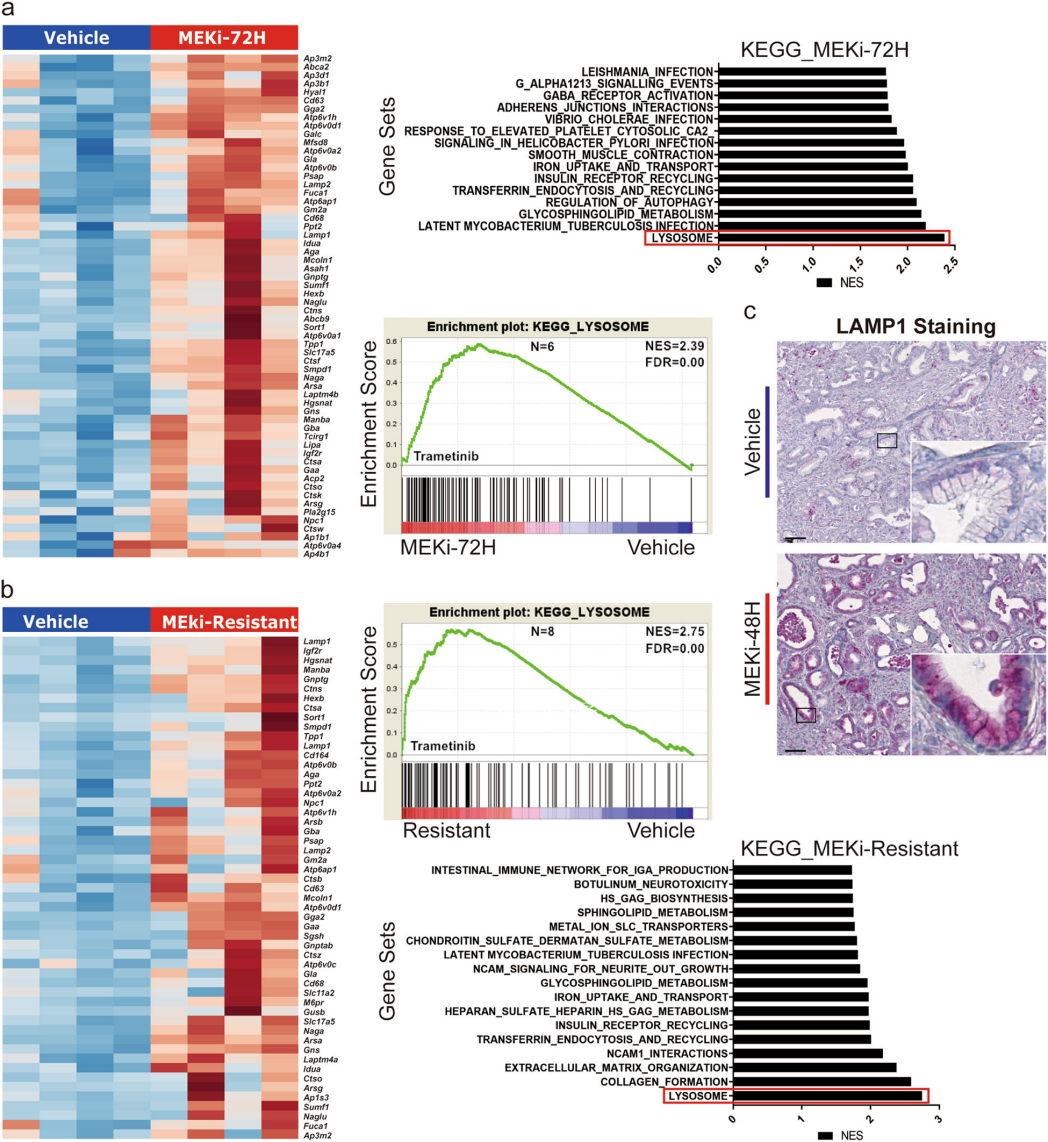
MEK inhibition activates expression of lysosome-associated genes and lysosomal biogenesis. **a** Heatmap of gene expression (RNA-seq) for core enriched lysosome-associated genes in 4 pairs of short-term trametinib-treated mouse PDAC cell lines (left panel) and KEGG pathway enrichment plot for lysosomes and other enriched KEGG gene sets in short-term trametinib-treated mouse PDAC cell lines (right panel). **b** Heatmap of gene expression (RNA-seq) for lysosome-associated core enriched genes in 4 pairs of trametinib-resistant mouse PDAC cell lines (left panel) and KEGG pathway enrichment plot for lysosomes and other enriched KEGG gene sets in trametinib-resistant mouse PDAC cell lines. **c** Immunostaining of the lysosomal marker *LAMP1* in refamatinib-treated mouse PDAC tumors tissue and vehicle control.

Using mouse PDAC-derived cell lines and mouse PDAC models, we investigated the impact of MEK inhibition on lysosomal biogenesis. Cell lines from mouse PDAC models were treated with trametinib for a period of 48 h with single dose IC_50_ and trametinib-resistant mouse PDAC cell lines were generated by chronic exposure. Gene expression and gene set enrichment analyses (GSEA) comparing short-term or trametinib-resistant cells with vehicle control revealed an increase in the expression of lysosomal markers as well as lysosomal enzymes such as *MCOLN1, LAMP1, LAMP2, ATP6V0D1 ATP6V1H* (Fig. 1a, b). Immunostaining of the lysosomal marker *LAMP1* was performed on tumor sections from short-term refametinib-treated mouse PDAC tumors equally showed an overexpression of the *LAMP1* gene product upon MEKi treatment (Fig. 1c).

In summary, MEK inhibition with distinct small molecule inhibitors led to increased lysosomal biogenesis signatures in various in vitro and in vivo models of murine PDAC.

### MEKi-induced lysosomal biogenesis is time-dependent

**Fig. 2:**
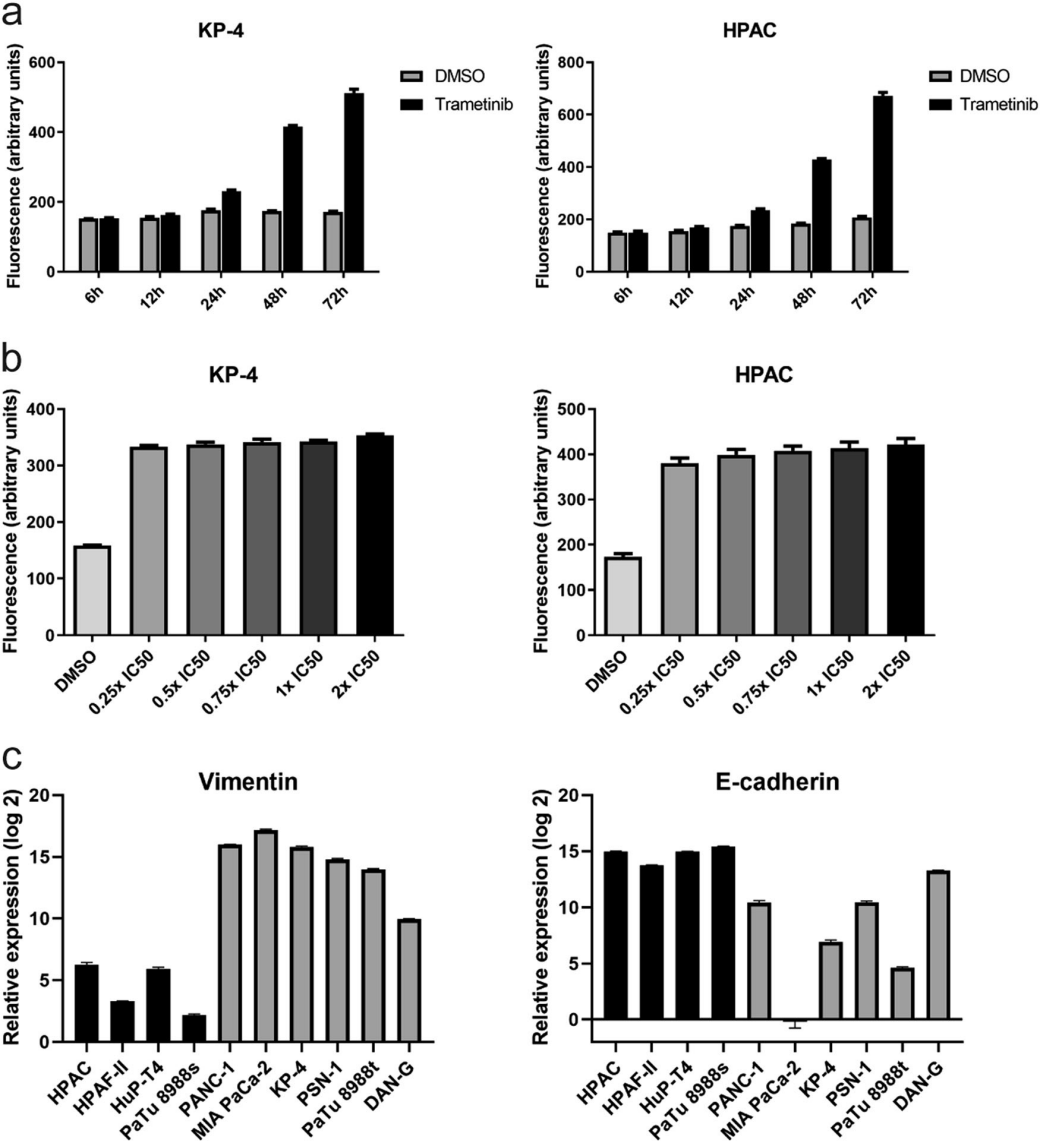
MEK inhibitor-induce lysosomal biogenesis is time dependent. **a** Time-dependent flow cytometric analysis of cellular lysosomal content upon trametinib treatment in two human PDAC cell lines (KP-4 and HPAC), three independent experiments each. **b** Concentration-dependent flow cytometric analysis of cellular lysosomal content in two human PDAC cell lines (KP-4 and HPAC) upon exposure to different IC_50_ concentrations of trametinib, three independent experiment. **c** Subtype characterization of human PDAC cell lines used in this study. Gene expression (qPCR, three independent experiments) for vimentin (left panel) and E-cadherin (right panel) was performed.

We next asked whether lysosomal biogenesis upon exposure to MEK inhibition is dose- and/or time-dependent. Exposure of human PDAC cell lines to single IC_50_ doses of trametinib for different time periods revealed a linear time-dependency in lysosomal levels, up to 72 h post-treatment (Fig. 2a). However, exposure of the same cell lines to increasing doses (from 0.25x IC_50_ to 2x IC_50_) of trametinib did not show any trend (Fig. 2b), indicating that drug concentration is less relevant for firing transcriptional programs responsible for increased lysosomal biogenesis. Given that PDAC constitute a heterogeneous disease with at least two major subtypes described, each having significant differences in therapy response and disease prognosis, we characterized our cell lines using the mesenchymal cell marker vimentin and the epithelial cell marker E-cadherin. All five epithelial human PDAC cell lines further used in the study had low vimentin expression and higher E-cadherin expression, meanwhile all mesenchymal cells had a higher vimentin expression and a lower E-cadherin expression (Fig. 2c). Cells were classified either as being of the classical or quasi-mesenchymal subtype based on previous classification^22^. All further analyses in this study were based on these cell lines.

### MEKi increases the lysosomal content and induces nuclear translocation of *TFEB*

**Fig. 3:**
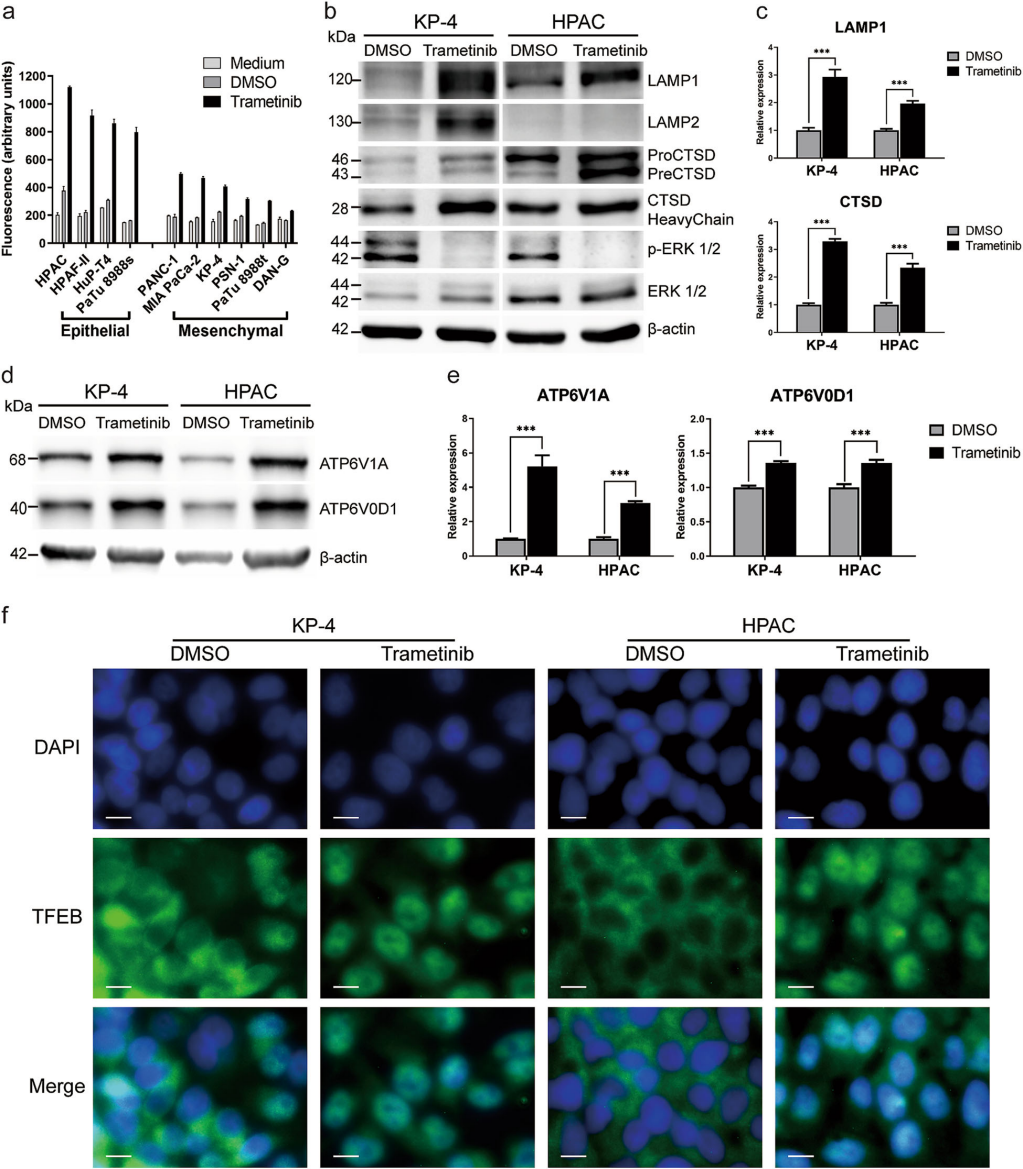
Trametinib treatment induces lysosomal biogenesis. **a** Fluorescent staining and flow cytometry analysis of cellular lysosomal levels in 10 different human PDAC cell lines (five classical and 5 mesenchymal) in three independent experiments. **b** Protein expression analysis for key lysosomal marker (*LAMP1, LAMP2, and CTSD*) in representative human PDAC cell lines with and without exposure to trametinib. Blot is representative of two independent experiments. **c** Transcript expression quantification (qPCR) of the lysosomal marker *LAMP1* (upper panel and the lysosomal hydrolase *CTSD* (lower panel) in two representative human PDAC cell lines. triplicates of two independent experiments are shown. **d**, **e** representative protein expression blot and transcript expression (qPCR) of lysosmal ATPases (*ATPV0D1* & *ATP6V1A*), respectively with and without trametinib exposure in two representative human PDAC cell lines. **f** Immunofluorescence staining of the lysosomal master regulator TFEB in two human PDAC cell lines with and without trametinib treatment. Images are representative of 2 independent stainings.

We next investigated the association between the observed increase in the expression of lysosomal-associated genes and cellular lysosomal levels. Using a fluorescent lysosome-specific dye, lysosomal content was measured by flow cytometry. As shown in Fig. 3a, cellular lysosomal levels were significantly increased in the trametinib-treated group compared with the vehicle control and medium-only groups. Unexpectedly, there was a stronger increase in lysosomal levels in PDAC cell lines related to the classical or epithelial (cl) subtype (HPAC, Hup-T4, Patu8988S, HPAF-II) compared with quasi-mesenchymal or basal-like (qm) PDAC cell lines (MiaPaCa-2, PSN-1, Patu8988T, KP4 and Panc-1) upon MEKi treatment (Fig. 3a). Upon trametinib treatment, an increase in the protein (Fig. 3b) and transcript (Fig. 2c) expression of lysosomal markers *LAMP1* and *LAMP2* as well as the lysosomal hydrolase *CTSD*. Furthermore, significant increases in the expression of direct *TFEB* targets such as *ATPV1A*, *ATPV0D1* (Fig. 3d, e and Supplementary Fig. 2) as well as other lysosomal ATPases and *TFEB* direct target genes such as *ATP6V0C, ATP6V1B2* and *CREG1* (Supplementary Fig. 1a). Concomitantly, increase in cellular lysosomal levels were associated with cytoplasmic to nuclear translocation of *TFEB* as revealed by immunofluorescence staining (Fig. 3f and Supplementary Figs. 3 and 4). In the qm PDAC subtype, nuclear *TFEB* was present in the vehicle control group and increased upon treatment, as opposed to the cl subtype, where *TFEB* was exclusively cytoplasmic in the vehicle control group.

### TFEB silencing inhibits Trametinib-induced lysosomal biogenesis

**Fig. 4:**
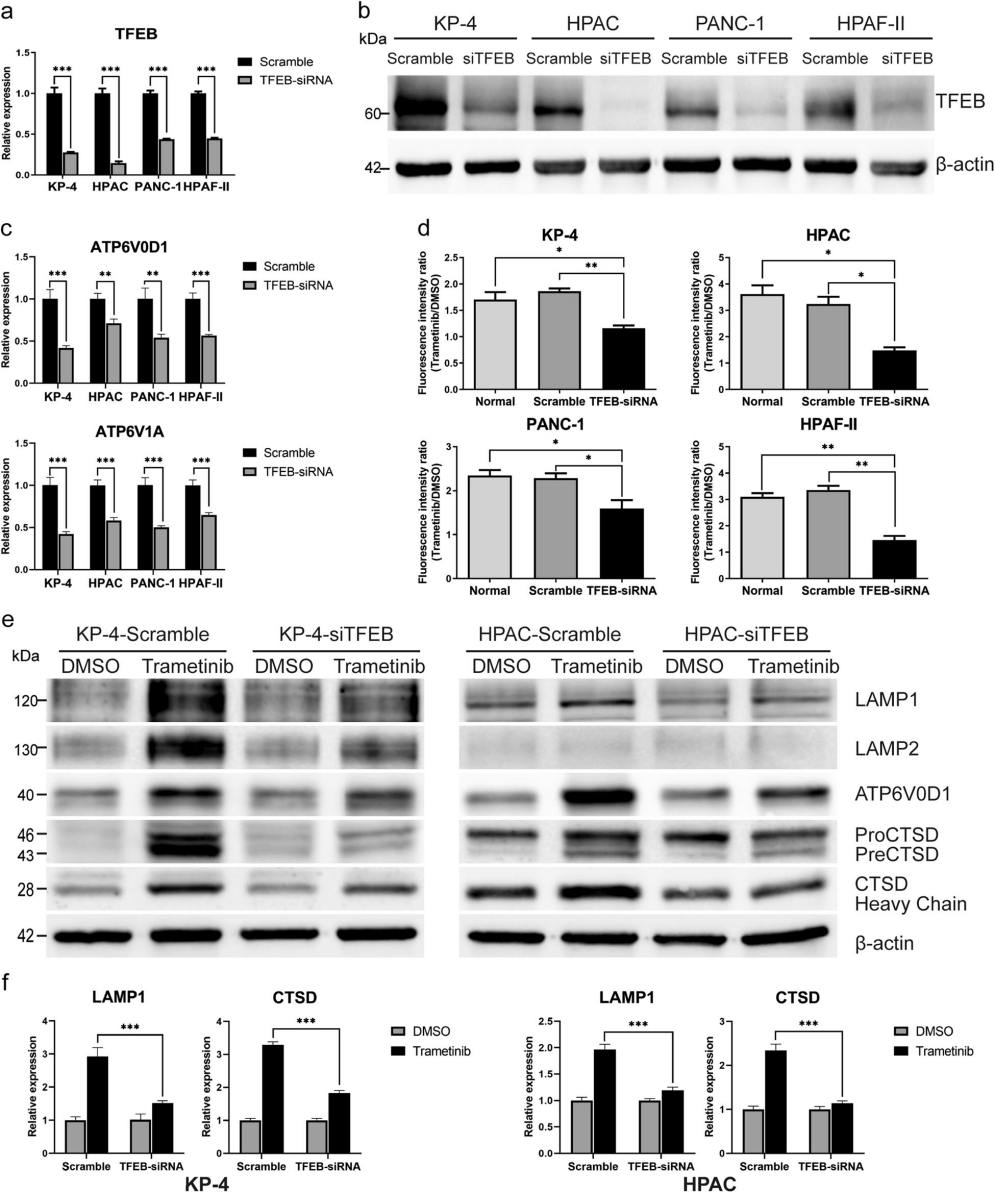
*TFEB* knockdown blocks trametinib-induced lysosomal biogenesis in human PDAC cell lines. **a**, **b** TFEB transcript expression (**a**) and protein expression (**b**), for the best targeting and non-targeting siRNA constructs in 4 human PDAC cell lines (*N* = 3). **c** Transcript expression of two direct *TFEB* targets (*ATP6V0D1* & *ATP6V1A*) upon *TFEB* knockdown in 4 human PDAC cell lines (*N* = 3). **d** Cellular lysosomal levels in *TFEB* knockdown cells and non-targeting control with and without exposure to trametinib (*N* = 3). **e**, **f** protein blot (**e**) and transcript expression (**f**) of major lysosomal genes and in *TFEB* knockdown cell with and without exposure to the MEK inhibitor trametinib. Reduced ERK1/2 phosphorylation as a read out for MEK inhibition is presented in Supplementary Fig. 4c.The same ß-actin loading control is used in bot blots.

To investigate the association between MEKi-induced lysosomal biogenesis and nuclear translocation of *TFEB*, we performed siRNA-mediated *TFEB* silencing. Four siRNA contructs targeting the *TFEB* locus (Supplementary Table 1) were evaluated and the best performing construct selected (Supplementary Fig. 4). Using the best-performing siRNA construct, *TFEB* knockdown was performed in 4 PDAC cell lines (2 cl and 2 qm). In all 4 human PDAC cell lines, a knockdown of at least 60% was achievable (Fig. 4a) and the protein expression was equally downregulated (Fig. 4b). Furthermore, the expression of direct TFEB target genes was significantly reduced (Fig. 4c and Supplementary Fig. 1b). As shown in Fig. 4d, downregulation of *TFEB* led to stabilized lysosomal levels upon MEKi treatment in both cl and qm-type PDAC cell lines. Indeed, fluorescent lysosomal staining of the *TFEB*-depleted cells after trametinib treatment did not show any significant increase in lysosomal contents as was observed in wildtype cells or cells transfected with a non-targeting siRNA. Both transcript and protein levels of lysosomal markers *LAMP1* and *LAMP2* as well as lysosomal-associated ATPases such as v*ATP6V0D1* and the hydrolase *CTSD* remained stable in the MEKi-treated group after *TFEB* knockdown compared with cell transfected with a non-targeting control (Fig. 4e, f).

### Trametinib accumulates in lysosomes and causes lysosomal exocytosis

**Fig. 5:**
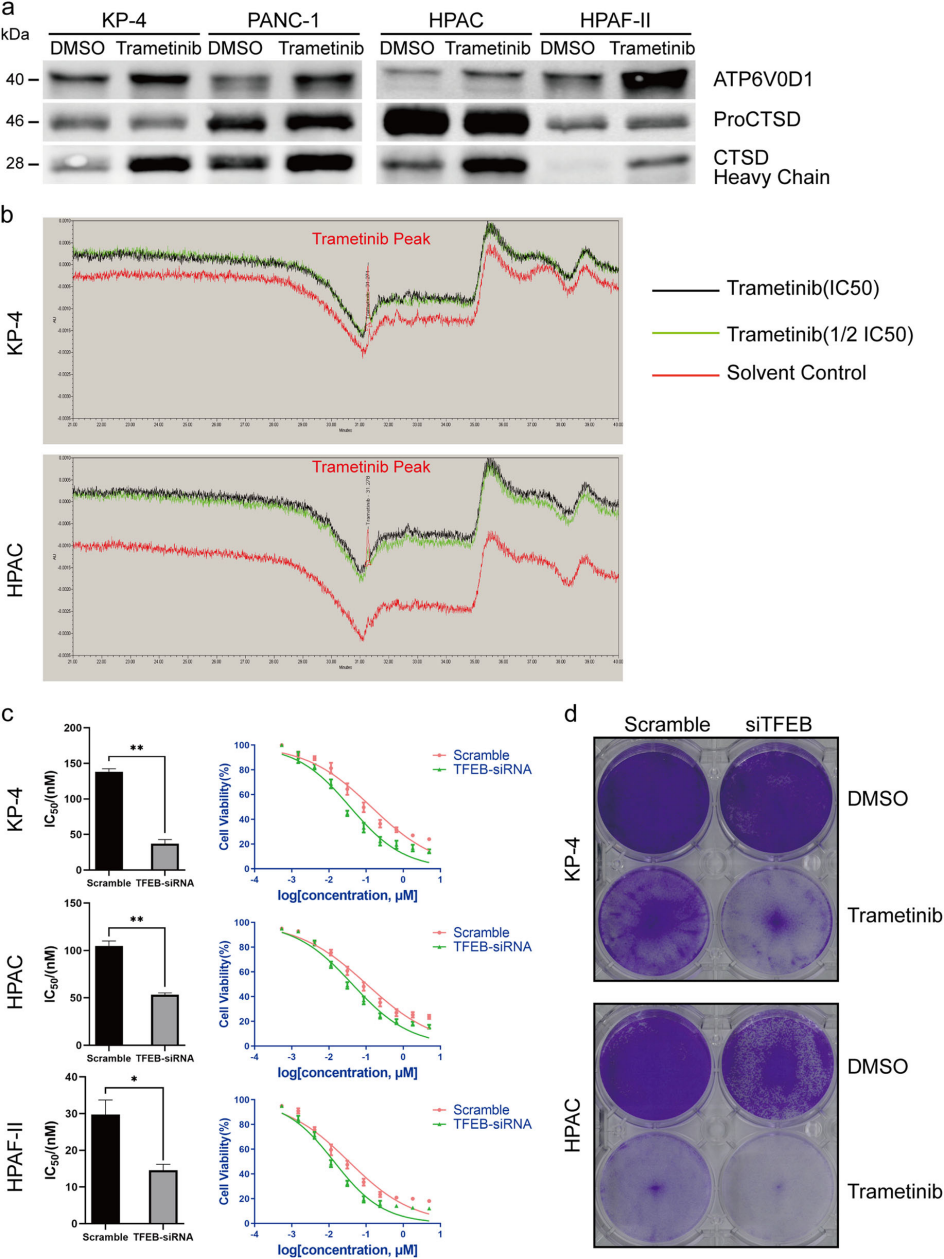
Trametinib accumulates in lysosomes and TFEB knockdown sensitized human PDAC cell lines to MEK inhibition. **a** Protein expression blot for lysosomal protein in cell culture supernatants from trametinib-treated and vehicle-treated human PDAC cell lines. **b** Mass spectrometer peak showing trametinib in lysosomes from human PAC cells treated with different doses of trametinib for 72 h (*N* = 2). **c** IC_50_ measurement (by means of cell titer glo assay) for trametinib in TFEB knockdown and scramble cells. **d** Colony formation assay performed on human PDAC cell after TFEB knockdown and exposure to trametinib or vehicle control.

Weak base anticancer drugs can be trapped in the lysosomes by cation trapping^16^. We next wanted to determine if increased cellular lysosomal levels could lead to drug sequestration. To determine if trametinib undergoes lysosomal trapping and possible excretion, cells were treated with a single IC_50_ dose of trametinib and lysosomes were isolated to directly quantify the amount of trametinib within the isolated lysosomes. As shown in Supplementary Fig. 6, the isolated cellular fraction was highly enriched for the lysosomal marker *LAMP2* and the lysosomal vATPase *ATP6V0D1*. After sonication to lyse the lysosomes and liberate their content, the lysosomal content was assessed for the presence of trametinib by LC-MS. Fig. 5b and Supplementary Fig. 7c show the mass spectra of trametinib from the isolated lysosomes and the trametinib standard respectively, indicating the presence of the drug in these organelles. We then investigated if the sequestered drug was disposed of in the extracellular milieu. We collected cell culture supernatant from trametinib-treated cells and isolated the protein fraction by ultrafiltration. This fraction was analyzed by western blot as shown in Fig. 5a. In effect, there were higher levels of the lysosomal hydrolase *CTSD* and the ATPase *ATP6V0D1* in cell culture supernatants from trametinib-treated cells compared with the vehicle control group. This suggest that lysosomal contents is being emptied in the extracellular milieu, although cell death could contribute partially as well.

### TFEB silencing sensitizes PDAC cells to MEK inhibition

Given the role of *TFEB* in MEKi-induced lysosomal biogenesis and the observation that trametinib is sequestered into the lysosomes and potentially excreted by lysosomal exocytosis, we asked whether *TFEB* knockdown would sensitize the cells to MEK inhibition. Indeed, after *TFEB* knockdown all tested cell lines were more sensitive to trametinib treatment as seen in the corresponding IC_50_ values (Fig. 5c). We performed a colony formation assay in MEKi-treated vs vehicle-treated *TFEB* knockdown and scramble cells. As shown in Fig. 5d, although *TFEB* knockdown affected the viability of the classical cell line HPAC in the absence of trametinib, no such difference was observed in the qm PDAC cell line KP4. However, upon trametinib treatment, cell viability was strongly affected in both cell lines, with a more pronounced effect observed in the *TFEB* knockdown group of the qm PDAC cell line KP4.

### MEK inhibition triggers lysosomal biogenesis in human PDAC in vivo

**Fig. 6:**
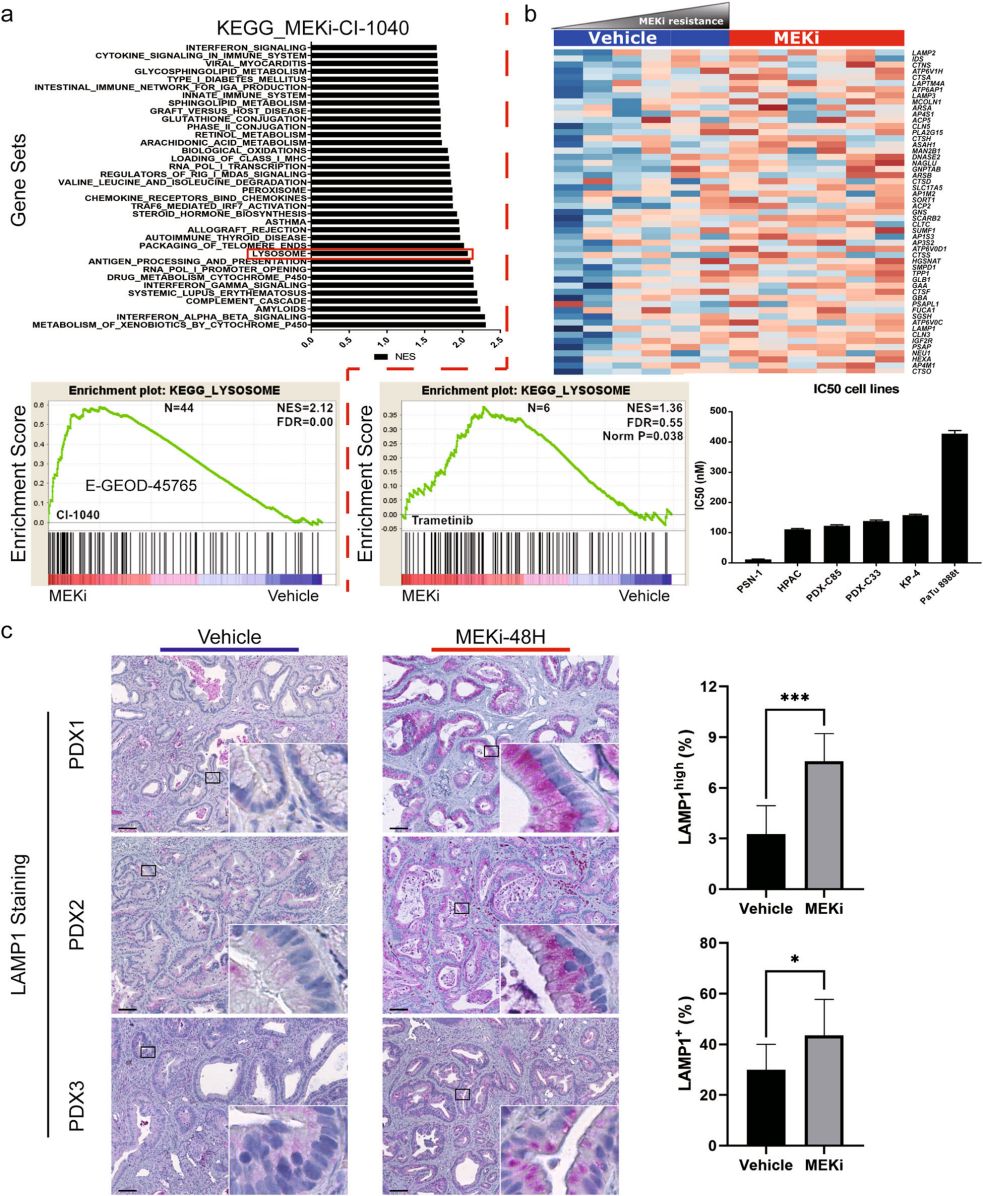
MEK inhibition induces lysosomal biogenesis in human PDAC xenografts. **a** KEEG gene set enrichment plot for enriched gene sets upon exposure to the MEK inhibitor CI-1040 (from publicly available data set, GSE 45765). **b** heatmap and KEGG pathway enrichment plot for human PDAC cell lines exposed to trametinib and IC_50_ values for human cell lines analyzed by RNA-seq (*N* = 2). **c** Immunostaining of the lysosomal marker LAMP1 in MEKi-treated human PDAC xenografts tissues with exposure to trametinib or vehicle control (left panel, *N* = 3) and IHC quantification of signal intensity (right panel).

Finally, we assessed whether MEKi-induced lysosomal biogenesis occurs in human PDAC in vivo. To test this hypothesis, we first performed gene expression analyses on human PDAC cell lines treated with single dose IC_50_ of trametinib or vehicle control. Additionally, using publicly available gene expression profiling data from human PDAC cell lines treated with a different MEK inhibitor (CI-1040) we performed Gene set enrichment analyses. In cell line treated with CI-1040, there was a strong enrichment in lysosomal gene set (Fig. 6a). Similarly, in cell line treated with the MEK inhibitor Trametinib, there was enrichment for lysosomal genesets, and the expression of core enriched genes increase with decreasine trametinib concentration (Fig. 6b). We then stained for the lysosome-specific marker *LAMP1* in trametinib-treated human PDAC xenograft tissues. Human PDAC PDX were generated and treated as described previously^23^. As shown in Fig. 6c and Supplementary Fig. 7b, human PDAC PDX models treated with the MEK inhibitor trametinib showed a significantly high expression of the lysosomal membrane marker *LAMP1* in the treated group compared with the vehicle group. The *TFEB* target gene *ATP6V1A* was equally highly expressed under MEKi treatment (Supplementary Fig. 7a).

## Discussion

The action mode of targeted agents is generally predicted through complex computer assisted modeling, followed by biological validation and molecular imaging^24–26^. However, intracellular drug interaction may also affect drug efficacy. In addition to adaptive resistance mechanisms such as drug efflux, activation of alternative oncogenic pathway, mutations development, and other factors such as selective drug sequestration may result in cellular unresponsiveness^27–29^. Lysosomal drug sequestration accounts for multi-drug resistance to several targeted therapeutic agents in cancer^30,31^.

We investigated MEKi-induced lysosomal drug accumulation in vivo and in vitro using human and mouse PDAC models to understand if drug sequestration could partly explain resistance observed to very potent MEK inhibitors such as trametinib. Treatment of PDAC tumors and cells with different MEK inhibitors led to increased lysosomal content and a consequent upregulation of lysosomal makers and lysosome-associated genes. Additionally, nuclear translocation of *TFEB* was observed after MEK inhibition and a corresponding upregulation of its target genes. This may point to the fact that MEK inhibition in PDAC induce lysosomal biogenesis and foster lysosomal drug accumulation and subsequent exocytosis as previously hypothesized^32^. To verify this hypothesis, we isolated lysosomes from trametinib-treated cells and using mass spectrometry,we were able to detect the presence of trametinib in these organelle.

Lipophilic amines with a pKa between 6.5 and 11 tend to accumulate more in a concentration-dependent manner in lysosomes^33^. Additionally, some anticancer drugs such as Gefitinib and Lapatinib (with a pKa of 4.5 and 3.8, respectively) are also trapped in lysosomes^34^. Irrespective of their acid-base properties, cells may sequester certain xenobiotics into the lysosomes as a detox mechanism. In fact, lysosomal drug sequestration plays a protective role in brain cells where lysosomes trap drugs and deposit them in neutrophils by extrusion^35^. Malignant cancer cells may adopt such mechanisms to guard against therapeutic assaults. Indeed, lysosomal drug sequestration has been reported for other anticancer agents^36^ and is responsible, at least in part for drug resistance^32,37,38^.

In human PDAC cell lines, we observed a strong increase in lysosomal levels in clPDAC cells compared to qmPDAC cells. In qmPDAC cells, *TFEB* was already present in the nucleus in basal conditions, as opposed to clPDAC cells, in which nuclear *TFEB* was observed only upon MEKi treatment. The presence of nuclear *TFEB* in qmPDAC cells before treatment may suggest a pre-existing xenobiotic sequestration and extrusion mechanism that accounts for the high resistance capabilities observed in these cells. This hypothesis is strengthened by the observation that a mesenchymal or basal-like phenotype is associated with poor outcome both in pancreatic and breast tumors^39,40^. Additionally, siRNA-mediated depletion of *TFEB* led to a more pronounced sensitization of qmPDAC cells to MEK inhibition compared to clPDAC cells. In fact, a 3-fold increase in MEKi sensitivity was observed in qmPDAC cells as opposed to a 1.5–2-fold increase in clPDAC cells. Lysosomal drug trafficking may therefore be contributing at least partially to intrinsic and acquired resistance to MEK inhibition in PDAC. This is further supported by the observation of a linear relationship between MEKi sensitivity and transcript abundance of lysosome-associated genes in vehicle-treated PDAC cells.

Accumulation of MEK inhibitors in intracellular lysosomes was associated with increased levels of lysosomal enzymes in cell culture supernatants and the presence of MEK inhibitor in intracellular lysosomes. Previous studies have reported *TFEB*-mediated lysosomal biogenesis and exocytosis upon treatment with different anti-cancer drugs^16^. Lysosomal targeting equally potentiates the activity of nintedanib in the treatment of non-small cell lung cancer^27^. Besides MEK inhibitor accumulation and exocytosis presented here, other targeted anticancer drugs such as tyrosine kinase inhibitors have also been reported to be accumulated in lysosomes^31,41^. Lysosomal drug accumulation and subsequent exocytosis may reduce the amount of bioavailable molecule at its target site thereby reducing its efficacy.

## Conclusion

Taken together, our data provides evidence of lysosomal drug sequestration and exocytosis during MEK inhibition both in vivo and in vitro in PDAC. We also show, that qmPDAC cells are more dependent on *TFEB* for modulation of MEKi sensitivity and demonstrate the presence of the targeted agent, trametinib, inside the lysosomes, an observation that may partly explain resistance to trametinib in PDAC.

## Materials and methods

### Cell culture and treatment

PDAC cells were obtained from the American Type Culture Collection (ATCC). All Cells were cultured in Dulbecco’s Modified Eagle’s Medium (cat no. 41965062, Life Technologies, Darmstadt, Germany) supplemented with 10% FBS and 1% pen-strep in a humidified incubator at 37 °C with 5% CO_2_. For short-term MEK inhibition, cells were treated with a single IC_50_ dose of trametinib (cat no. SYN-1170-M010, Biomol, Hamburg, Germany) for 72/48 h (human cell lines/mouse cell lines) or an equal volume of vehicle control (DMSO). Long-term MEK inhibition in mouse PDAC cell lines was achieved by chronic exposure to increasing doses of trametinib up to 100x IC_50_. Four different siRNA targeting TFEB (Supplementary Table 1) were purchased from Qiagen. Transfections were performed using the HiPerFect transfection reagent (cat no. 301705, Qiagen, Hilden, Germany) according to the manufacturer’s instructions and analyzed by western blotting and qPCR.

### Mice and treatments

*Ptf1a*^wt/cre^; *Kras*^wt/LSL-KrasG12D^; *Tp53*^*fl/fl*^ (CKP) mice developing spontaneous aggressive PDAC were generated as previously described^42^ and treated for 48 h with the MEK inhibitor refametinib^43^. Patient-derived xenografts were generated by subcutaneous transplantation of resected PDAC tumors or from purified circulating tumorigenic cancer stem cells in NOD/SCID/IL2y- mice and propagated in NMRI:nu/nu mice after engraftment^23^. After morphological and molecular characterization, animals were treated with a single dose of trametinib (1 mg/kg) as previously reported^23^.

### RNA sequencing and gene set enrichment analyses

Total RNA was isolated using the Maxwell RSC simplyRNA Cells Kit (cat no. AS1390, Promega, Fitchburg, USA) following manufacturer’s instruction. 100 ng of total RNA was used for library preparation using the TruSeq Stranded mRNA (cat no. 20020595, Illumina, San Diego, USA) and subjected to 100 bp paired end sequencing on a HiSeq 4000. After quality control with FastQC, sequencing reads were trimmed and mapped to the human genome (Genome 38) with STAR and features were quantified using htSeq count. Additionally, publicly available data from MEKi-treated PDAC cell lines was downloaded from the gene expression omnibus (GSE-45765)^44^.

Gene set enrichment analyses (GSEA) was performed using the Broad Institute algorithm with gene set permutation on the Hallmarks, KEGG, Reactome and Gene ontology gene sets. Significance cut off was set to a false discovery of <0.05 (Benjamini & Hochberg).

### Lysosomal staining and flow cytometry

For lysosomal staining, cells were grown to about 70% confluence and then incubated with Cytopainter LysoGreen indicator reagent (1:5000, cat no. ab176826, Cambridge Biomedical Campus, Cambridge, UK) for 1 h at 37 °C with 5% CO_2_. Cells were then washed twice in pre-warmed Hank’s balanced salt solution (HBSS, cat no. 14175053, Life Technologies), trypsinized with 0.05% trypsin EDTA (cat no. 25300054, Life Technologies) and resuspended in 1x phosphate buffered saline (PBS, cat no. 14190169, Life Technologies) for flow cytometry. Lysosomal levels were measured by quantifying the dye fluorescence in a Beckman Coulter Gallios flow cytometer (Beckman Coulter, Brea, USA).

### Immunohistochemical staining and quantification (IHC)

Immunostaining was performed on 5 µM sections of formalin-fixed paraffin-embedded tissues using the Dako REAL Alkaline Phosphatase Detection System (cat no. K500511-2, Dako, Santa Clara, USA). Sections were dewaxed on an automated dewaxing system (Leica ST5010 Autostainer XL, Leica Geosystems, Heerbrugg, Switzerland) and heat-induced epitope retrieval was performed using citrate buffer (10 mM Sodium Citrate, 0.05% Tween 20, pH = 6) for 15 min at 110 °C in a pressure cooker. After blocking with serum-free protein blocking solution for 30 min (cat no. X0909, Dako), slides were incubated with a 1:250 dilution of primary antibodies at 4 °C overnight. The slides were then washed in TBS-T buffer 3 times for 5 min,incubated with secondary antibody for 30 min at room temperature, and then subjected to Fast Red chromogen development using permanent AP red kit (cat no. ZUC001-125, Zytomed Systems, Berlin, Germany). Slides were counterstained with hematoxylin, dehydrated, and mounted.

Slides were scanned and digitalized with a Zeiss Axio Scanner Z.1 (Carl Zeiss AG) at 2.5X and 10X magnification. The percentage of positive cells was quantified by Definiens software version 4.3.1 (Definiens AG) on the whole area of the pancreatic ductal cells per field. The total number of cells in each field was determined based on nuclei staining detected by the software.The average number of positive cells and high positive cells were expressed as a percentage of the total number of cells from each field.

### Immunofluorescence staining

Cells were grown on 8-well cell culture chamber slides (cat no. 154534, Lab-Tek, Waltham, USA). 3 days after treatment with a single IC_50_ dose of trametinib or DMSO control (cat no. D2438, Sigma-Aldrich, St. Louis, USA), slides were washed with PBS 3 times and fixed in 4% paraformaldehyde in PBS for 15 min. Slides were then washed with cold PBS 3 times for 5 min and permeabilized with 0.3% Triton-X100 in PBS for 20 min at room temperature. After washing as before, slides were blocked with 5% BSA in PBS for 45 min. Cells were incubated with a 1:100 dilution of the corresponding primary antibodies (Supplementary Table 2) in 1% BSA at 4 °C overnight. Slides were washed and incubated with the secondary antibody (1:400; cat N° SA5-10086, Thermo Scientific, Waltham, USA) for 1 h at room temperature in the dark. Afterwards, slides were washed 3 times with PBS, counterstained with DAPI (cat N° H-1200, Vector Laboratories, Burlingame, USA) and scanned with a Zeiss Scanner (40×).

### Cell viability (IC_50_) and colony formation assays

Trametinib was dispensed in white flat bottom 96 well plates in triplicate in a logarithmic range from 0.0015 µM to 5 µM, and 3000–4000 cells (cell line dependent optimal numbers, Supplementary Table 3) were seeded in the drug-containing plates using a multidrop (Multidrop Combi, Thermo Scientific). After 72 h, an equal volume of CellTiter-Glo reagent (cat N° G7573, Promega) was added, mixed vigorously by shaking for 2 min and incubated at room temperature for 10 min. Luminescence signal was measured on the Spark Multimode Microplate Reader (Tecan Group, Männedorf, Switzerland). Triplicates were averaged,dose response curves were analyzed in GraphPad Prism version 8 and IC_50_ values were determined.

For colony formation assays, cells were grown in 6-well plates to a 30%confluency and treated with a single IC_50_ drug dose or vehicle control for 72 h. Cells were then washed with PBS once at room temperature, fixed for 10 min in ice-cold methanol and then stained with crystal violet (cat no. C0775-25G, Sigma-Aldrich) for 30 min. Plates were washed twice with water and dried for 24 h.

### Secretome protein isolation

Cells were cultured to 75–85% confluence in a T175 flask. Cell culture medium was then removed and cells were washed 3 times with PBS followed by 2 times wash with serum-free growth medium. Subsequently, cells were treated with a single IC_50_ dose of trametinib or vehicle control for 48 h in serum-free growth medium. The cell culture supernatant was then collected and centrifuged at 500*g* for 5 min. The resulting supernatant was concentrated using 3 kDa molecular-weight cutoffs polyethersulfone membrane (cat no. PES-3K, Pierce Protein Concentrator, Thermo Scientific) by centrifugation at 3500xg in a swinging-bucket rotor at 4 °C for 3–4 h and stored at -20 °C.

### Lysosomal isolation

Lysosomal fractions were isolated from cultured cells by density gradient centrifugation in a Optima L-80 XP Ultracentrifuge (35,000 rpm, 2 h) using the Lysosome Enrichment Kit for Tissue and Cultured Cells (cat no. 89839, Thermo Scientific), according to the manufacturer’s protocol. The purity of the fractions was assessed by measuring expression of lysosome-specific protein by western blot. Accumulation of trametinib in lysosomes was analyzed by liquid chromatography coupled to mass spectrometry (LC-MS, Waters GmbH).

### Identification of trametinib in lysosomes

Lysosomal Trametinib was analyzed with a RP-HPLC (Waters GmbH, Eschborn, Germany). Analytes were separated on an EC250/4 Nucleodur C18 ISIS, 5 µm (cat no. 760414.40) with pre-column CC8/4 (cat. no. 761310.40, Macherey-Nagel, Düren, Germany) thermostatted at 40 °C. A gradient at a flow rate of 1.00 ml/min was achieved with mobile phase of A = formic acid (0.1%), B = methanol, and C = acetonitrile, respectively. Split ratio between PDA- and MS-detector was set to 85/15 (PDA/MS, vol/vol). Mass spectrometry was performed in positive ion electrospray ionization mode. Masses for the detection of Trametinib were optimized after extraction from total ion chromatogram.

### Western blot analysis

Proteins were extracted in 1x RIPA Buffer (cat no. 9806S, Cell Signaling Technologies, Danvers, USA) supplemented with 1x protease and phosphatase inhibitor cocktails (cat no. 5872, Cell Signaling technologies). Protein concentration was determined by BCA protein assay (cat no. 23225, Thermo Scientific). Samples were resolved on 8–10% SDS polyacrylamide gels and blotted on 0.2 µM nitrocellulose membranes (cat no. 1704270, Bio-Rad, Hercules, USA). Membranes were blocked with 5% BSA (in TBS-T) at room temperature for 1 h on an orbital shaker and then incubated with primary antibodies diluted in blocking buffer at 4 °C overnight. Secondary antibodies were diluted in blocking buffer and incubated at room temperature for 1.5 h. Membranes were developed with the SuperSignal Chemiluminescent (cat no. 34075, Thermo Scientific) and scanned with a ChemiDoc MP Imaging System (Bio-Rad).

### Real-time quantitative PCR

Total RNA was extracted using the Maxwell RSC simplyRNA Cells Kit (cat no. AS1390, Promega) according to the manufacturer’s instructions. cDNA was synthesized from 1 µg of total RNA using SuperScript IV First-Strand Synthesis System (cat no. 18091200, Thermo Scientific). Transcript quantification was performed using a 1x sybrgreen mix in 12 µl reactions on a Roche LightCycler 480 using Light Cycler 480 SYBR Green I Master Kit (cat no. 04887352001, Roche GmbH, Basel, Switzerland) Primer sequences used are listed in Supplementary Table 4.

## Supplementary information

Solvent Control_KP4_HPAC Paper Ben_Two different runs

Original ß-actin blot for figure 3b

Original ß-actin blot for figure 3d

Original ß-actin blot for figure 4b

Original ß-actin blot for figure 4e-1

Original ß-actin blot for figure 4e-2

LC-MS Figure 5b KP4 cell line

LC-MS Figure 5b HPAC cell line

supplementary Fig 1

supplementary Fig 2

supplementary Fig 3

supplementary Fig 4

supplementary Fig 5

supplementary Fig 6

supplementary Fig 7

Supplementary figures and tables

Author contribution
