# Supplementary material for: TFEB-mediated lysosomal biogenesis and lysosomal drug sequestration confer resistance to MEK inhibition in pancreatic cancer
Source: Cell Death Discov. 2020 Mar 11;6:12. doi: 10.1038/s41420-020-0246-7 (PMC7066197; doi:10.1038/s41420-020-0246-7)
Supplement: Supplementary file 1 — Solvent Control_KP4_HPAC Paper Ben_Two different runs [file 41420_2020_246_MOESM1_ESM.docx]

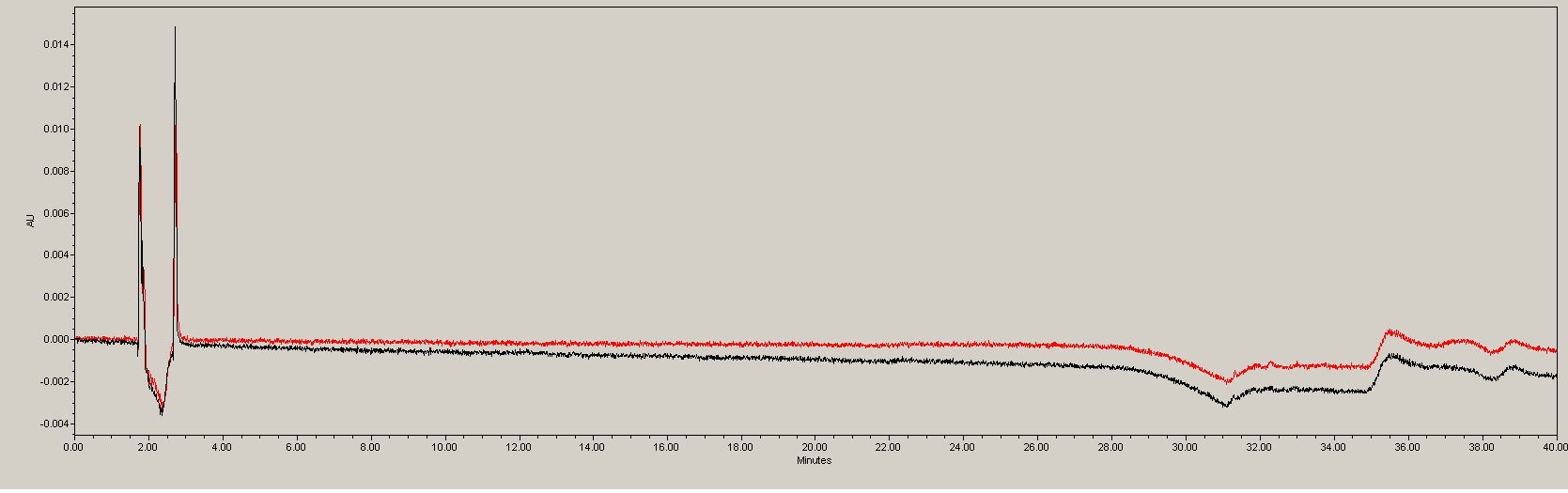


Solvent controls: Complete Chromatograms


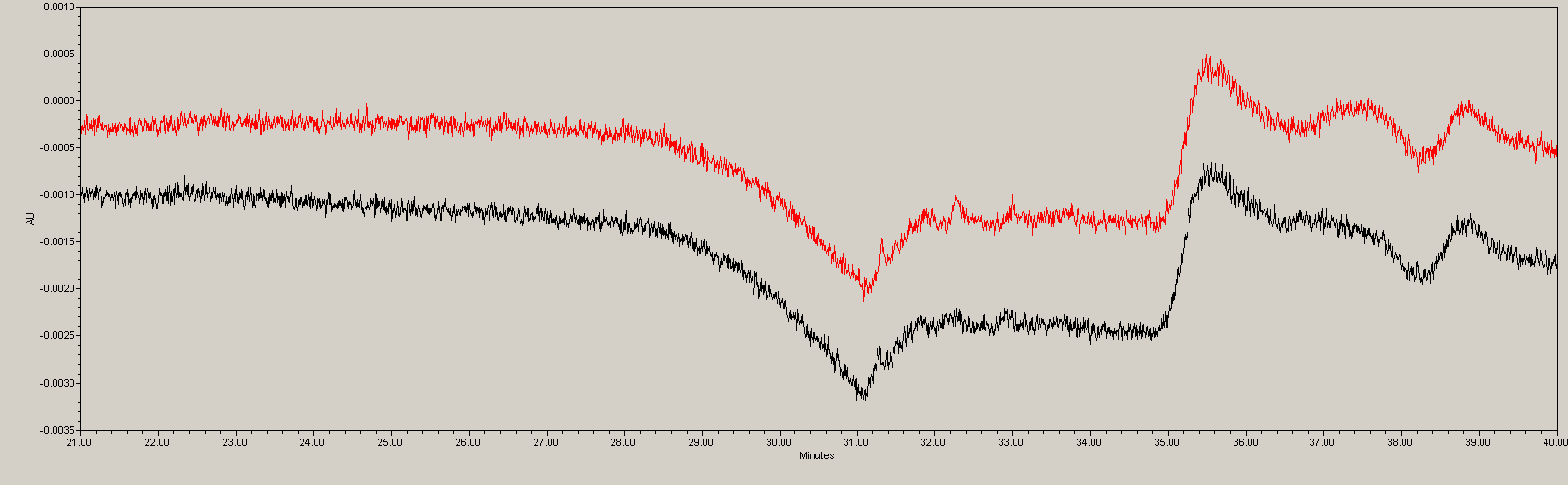


Zoom between retention time 21 and 40 minutes

Red line: Date Acquired 12/02/2019 07:42:23 CET

Black line: Date Acquired 25/02/2019 09:12:51 CET


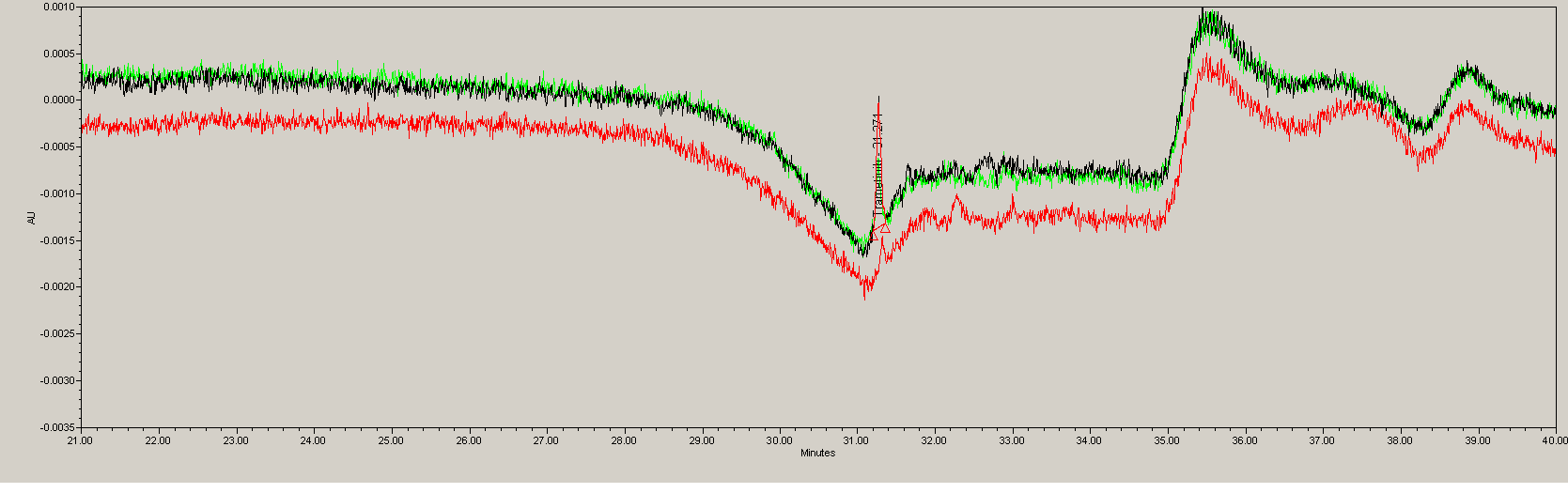


KP4: IC50 = black; 1/2IC50 = green; solvent control = red


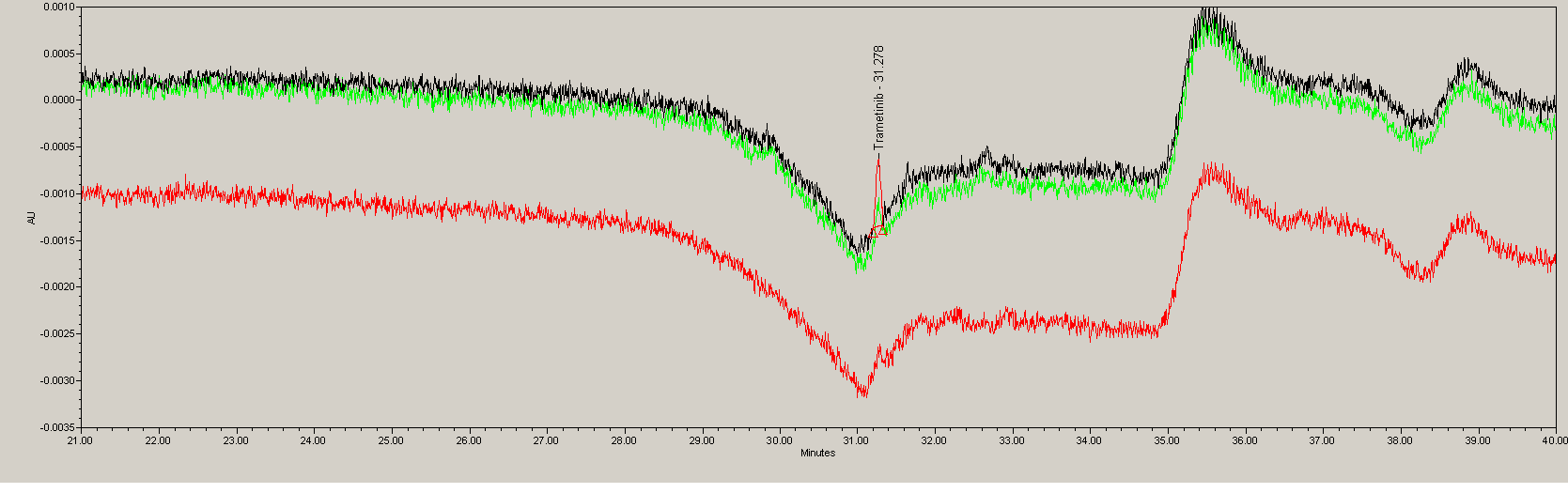


HPAC: IC50 = black; 1/2IC50 = green; solvent control = red
