# Supplementary figures and images for: TFEB-mediated lysosomal biogenesis and lysosomal drug sequestration confer resistance to MEK inhibition in pancreatic cancer
Source: Cell Death Discov. 2020 Mar 11;6:12. doi: 10.1038/s41420-020-0246-7 (PMC7066197; doi:10.1038/s41420-020-0246-7)

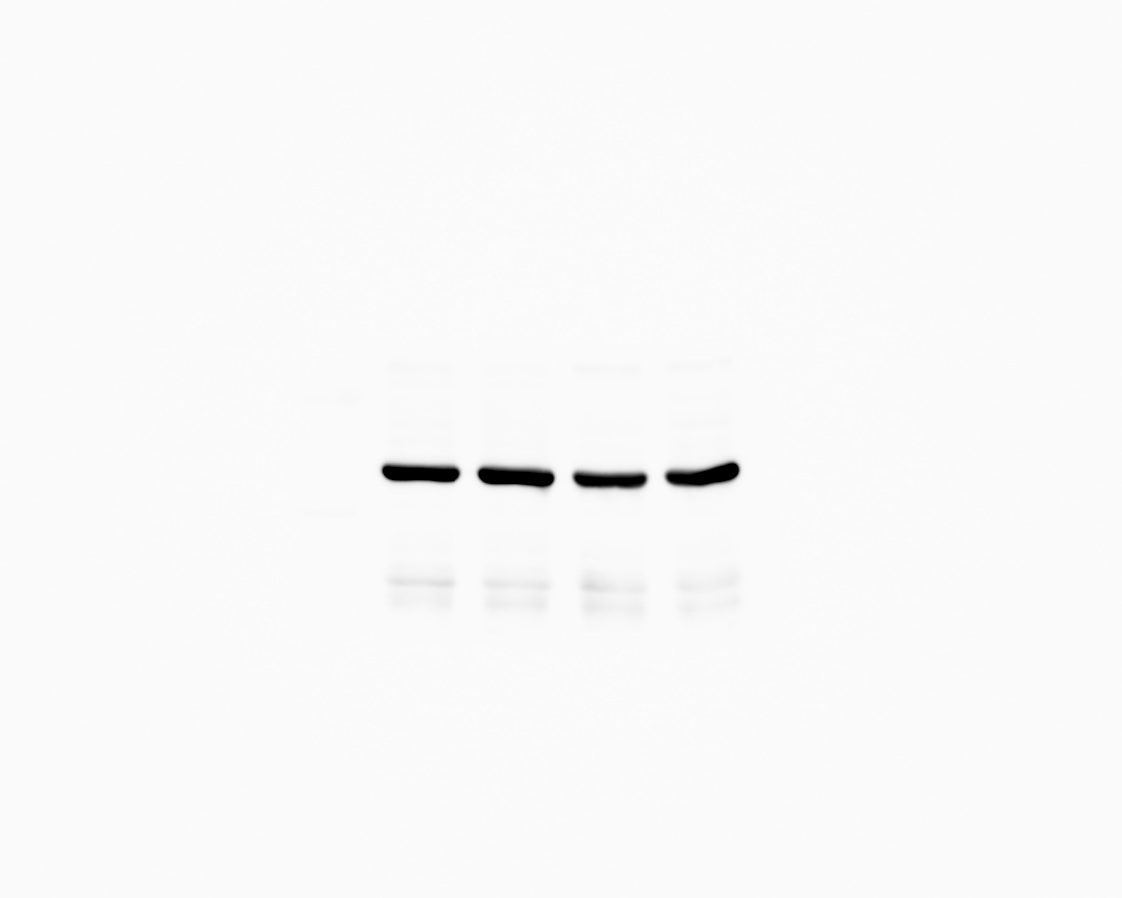

Supplement: Supplementary file 2 — Original ß-actin blot for figure 3b [file 41420_2020_246_MOESM2_ESM.tif]

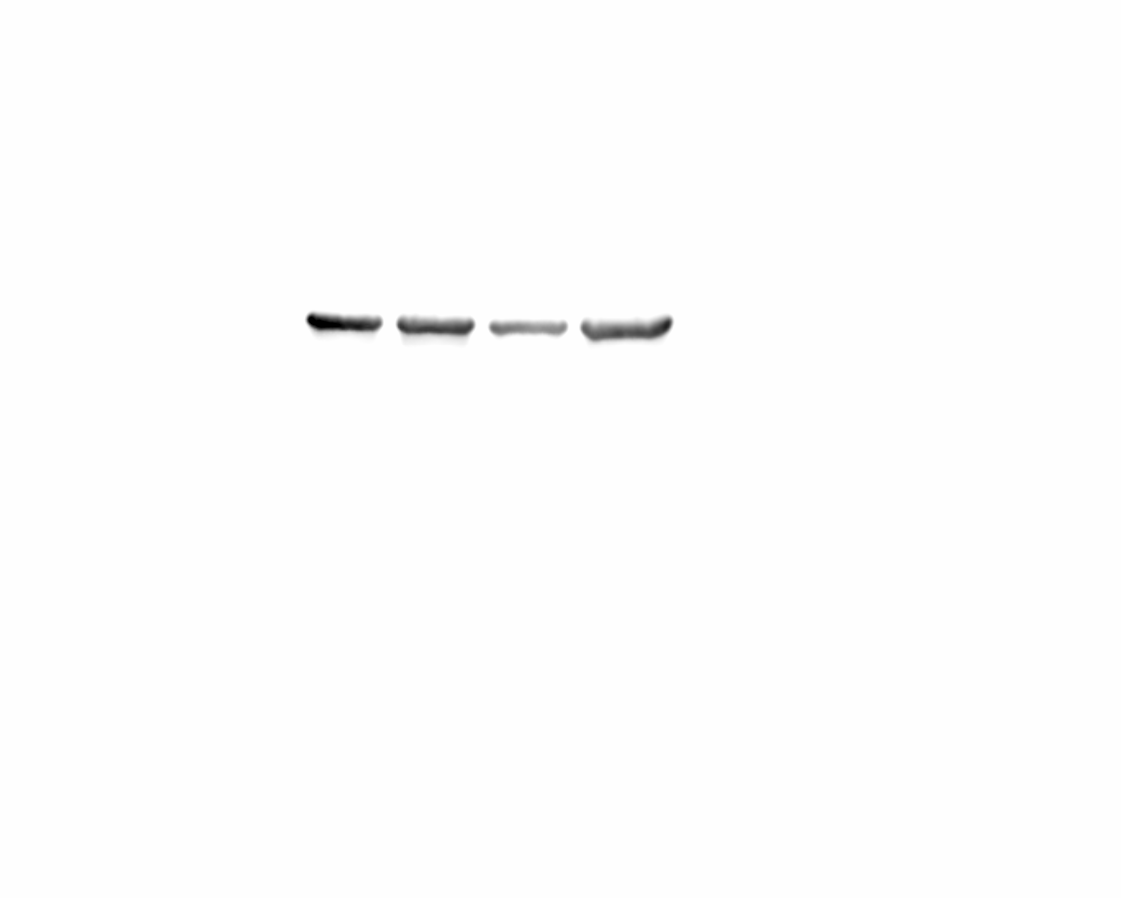

Supplement: Supplementary file 3 — Original ß-actin blot for figure 3d [file 41420_2020_246_MOESM3_ESM.tif]

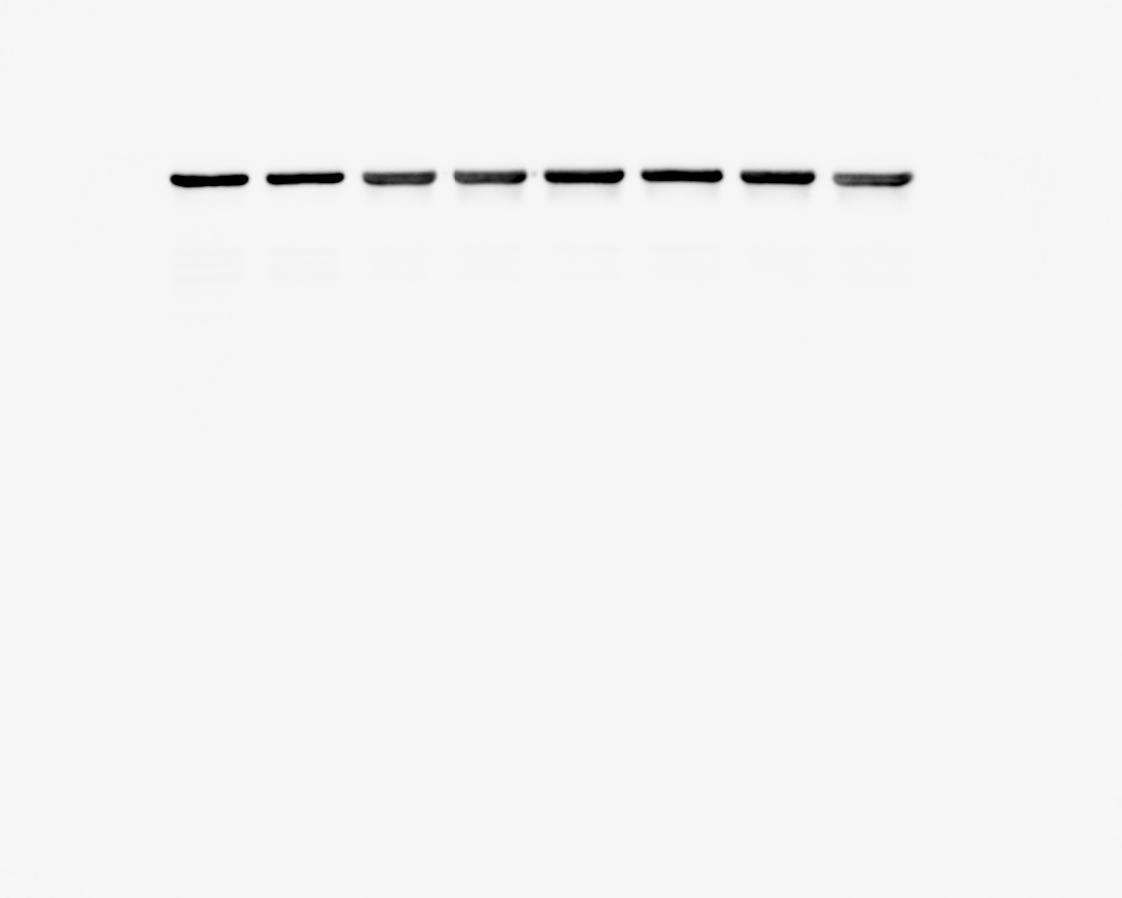

Supplement: Supplementary file 4 — Original ß-actin blot for figure 4b [file 41420_2020_246_MOESM4_ESM.tif]

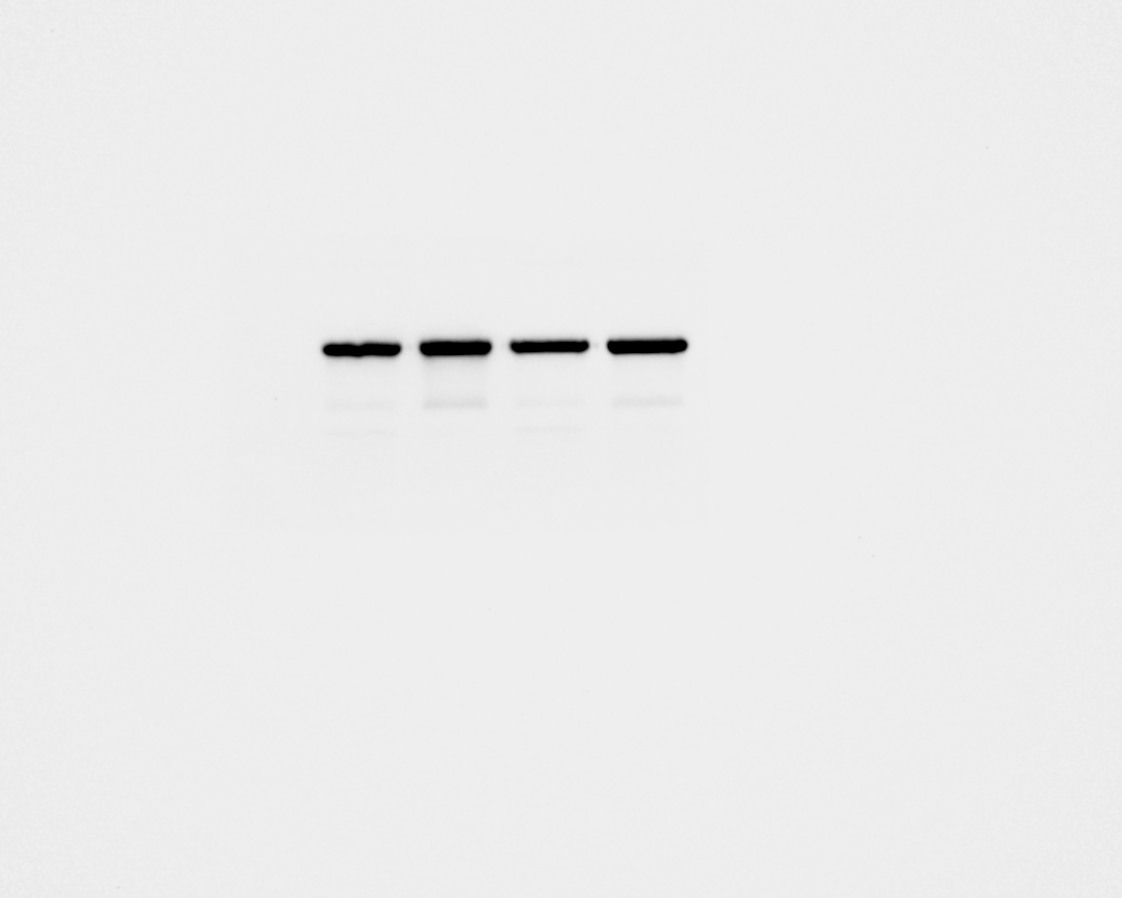

Supplement: Supplementary file 5 — Original ß-actin blot for figure 4e-1 [file 41420_2020_246_MOESM5_ESM.tif]

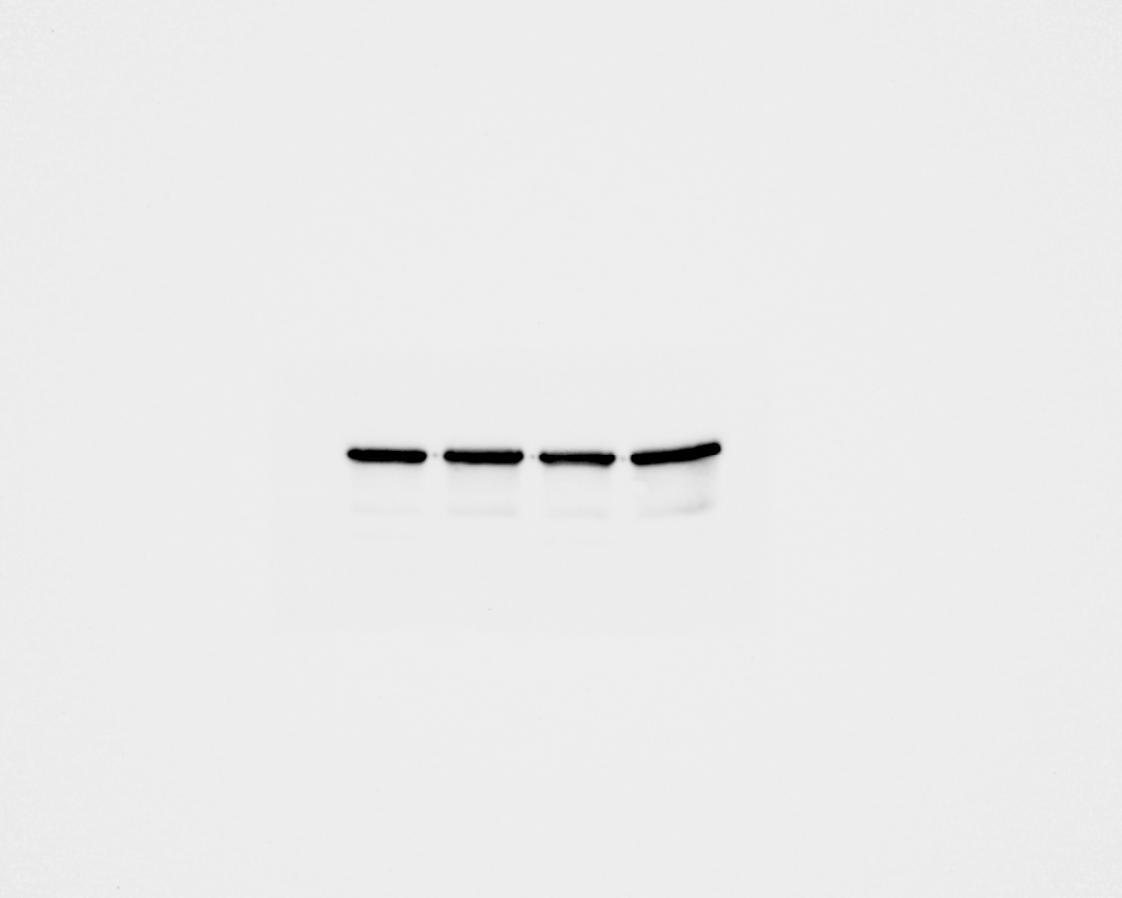

Supplement: Supplementary file 6 — Original ß-actin blot for figure 4e-2 [file 41420_2020_246_MOESM6_ESM.tif]

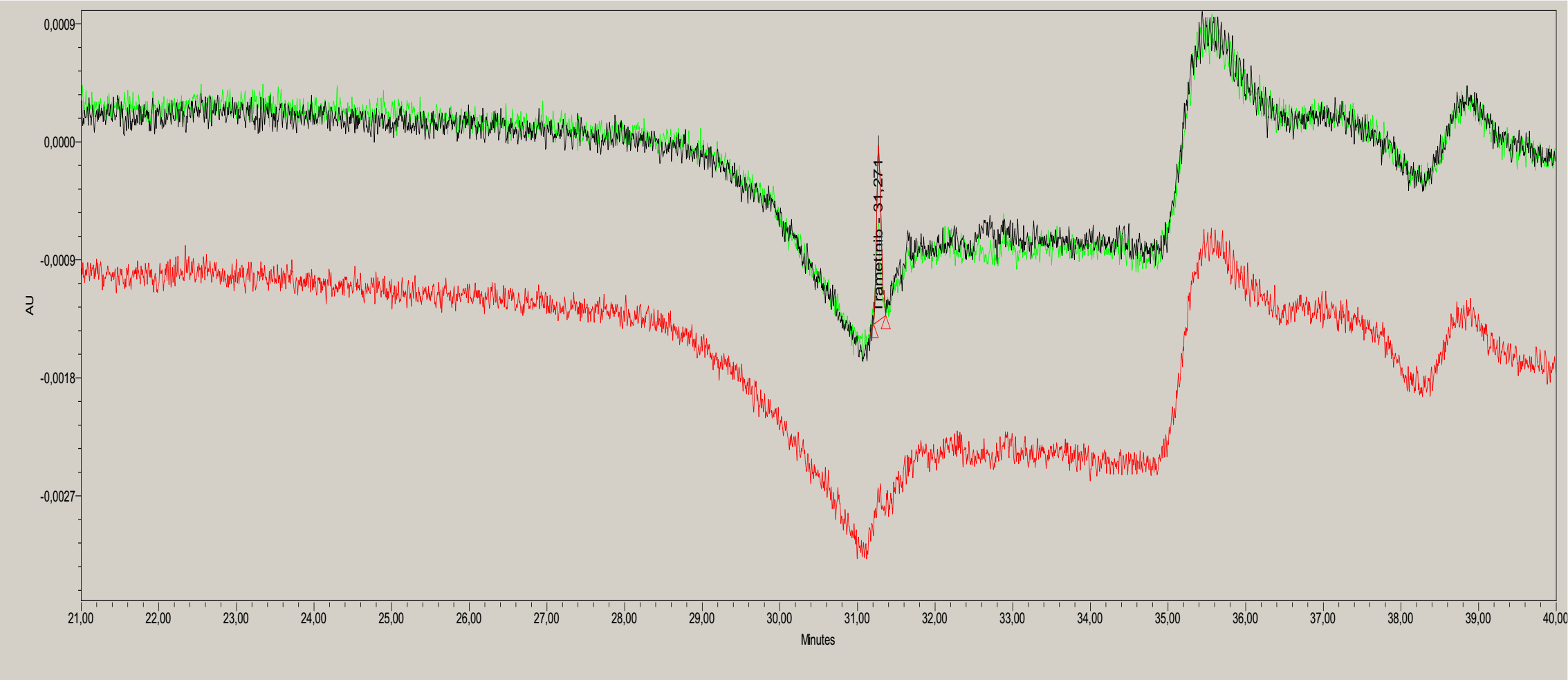

Supplement: Supplementary file 7 — LC-MS Figure 5b KP4 cell line [file 41420_2020_246_MOESM7_ESM.tif]

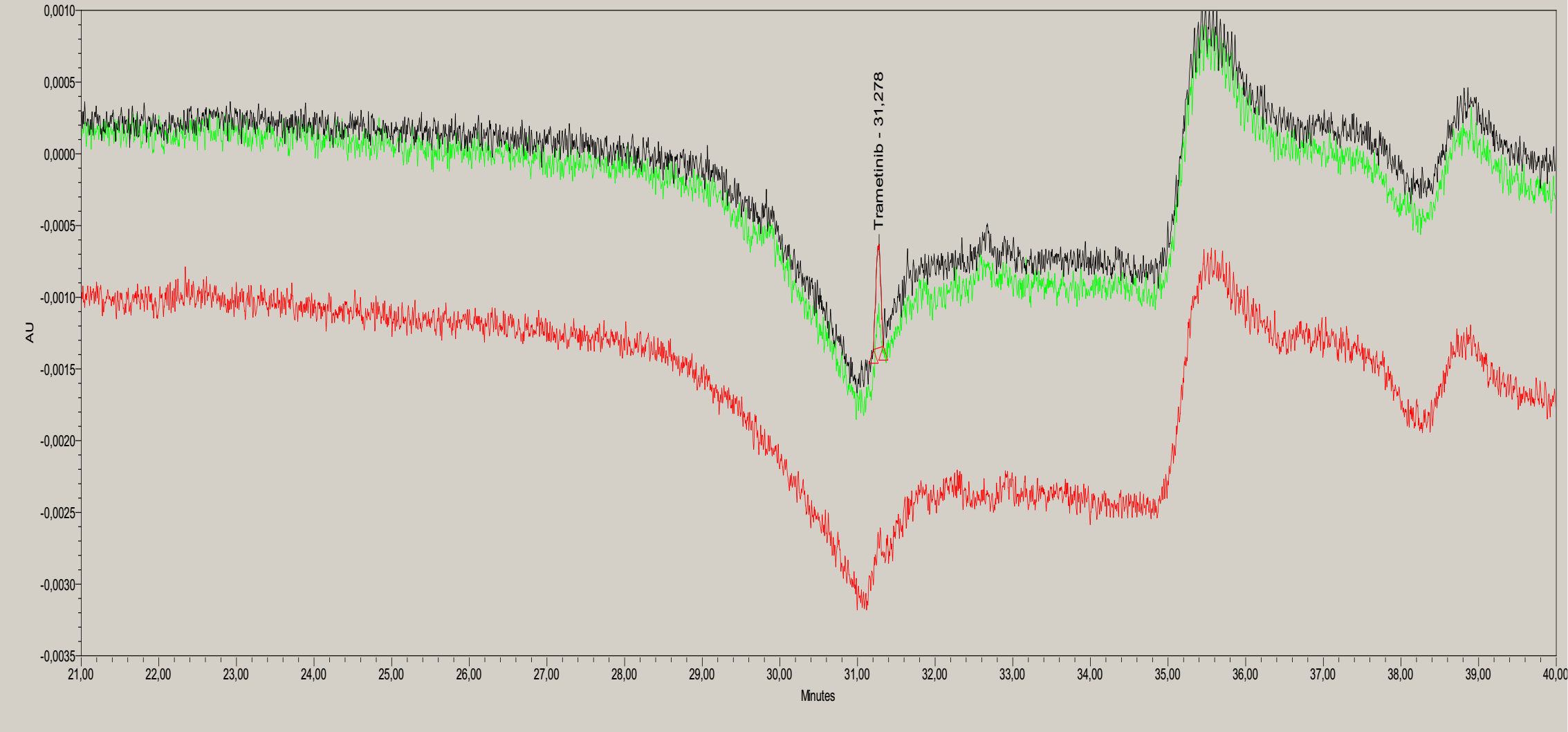

Supplement: Supplementary file 8 — LC-MS Figure 5b HPAC cell line [file 41420_2020_246_MOESM8_ESM.tif]

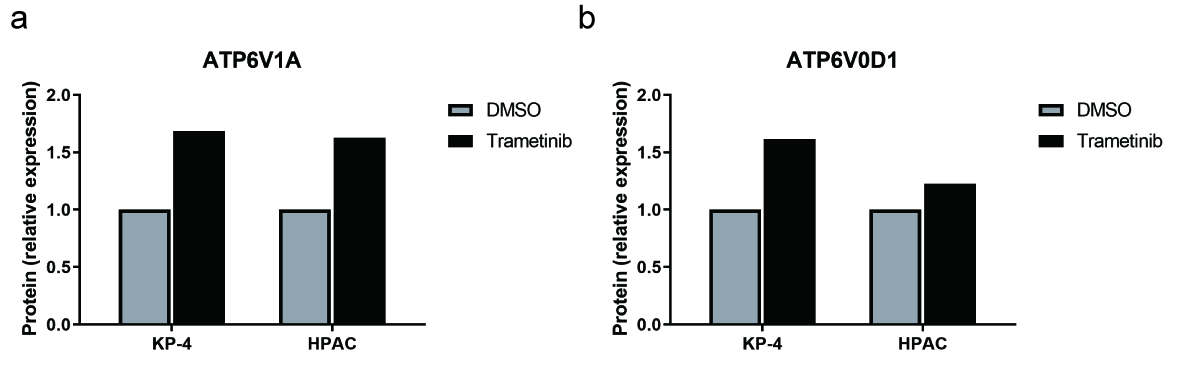

Supplement: Supplementary file 9 — supplementary Fig 1 [file 41420_2020_246_MOESM9_ESM.tif]

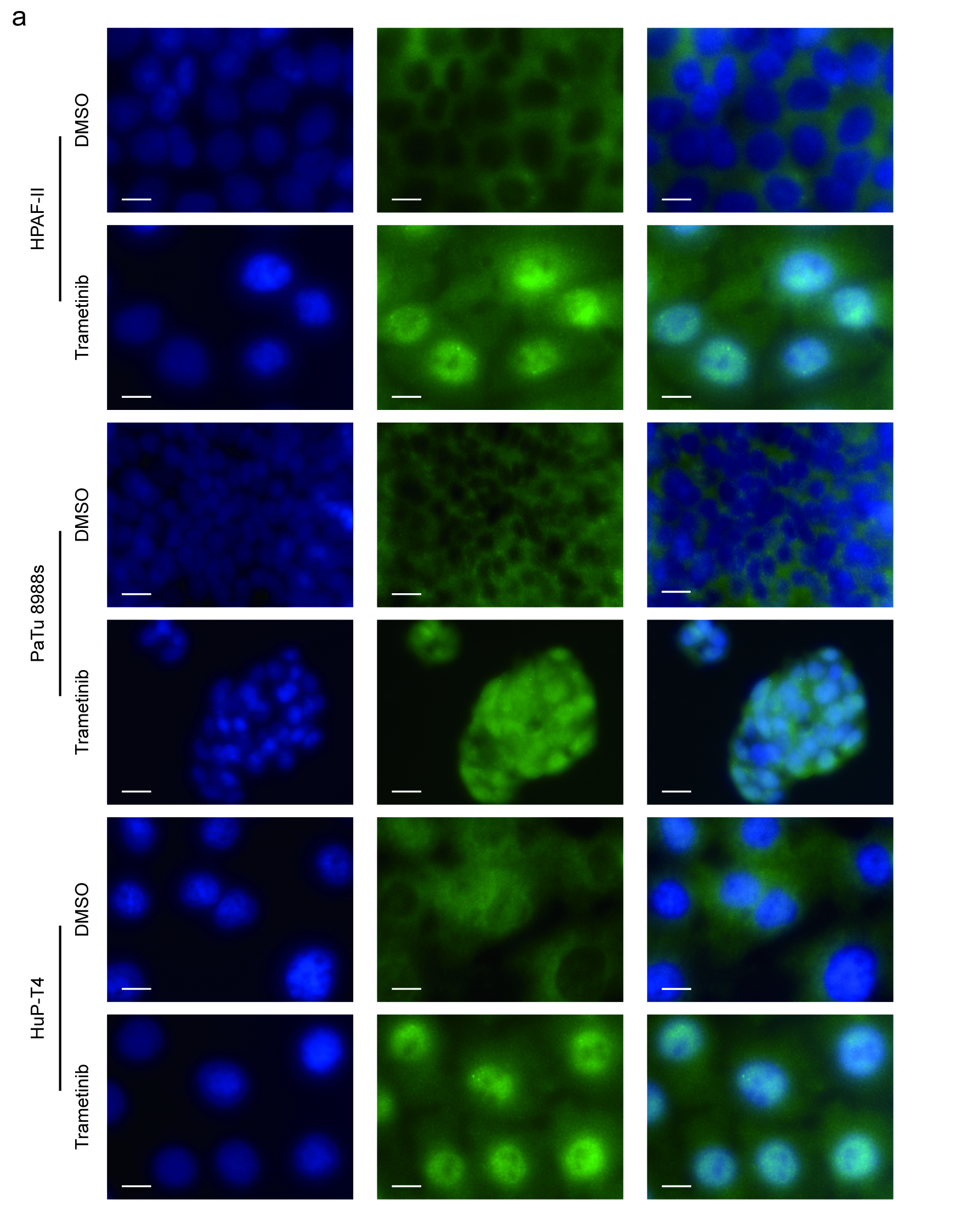

Supplement: Supplementary file 10 — supplementary Fig 2 [file 41420_2020_246_MOESM10_ESM.tif]

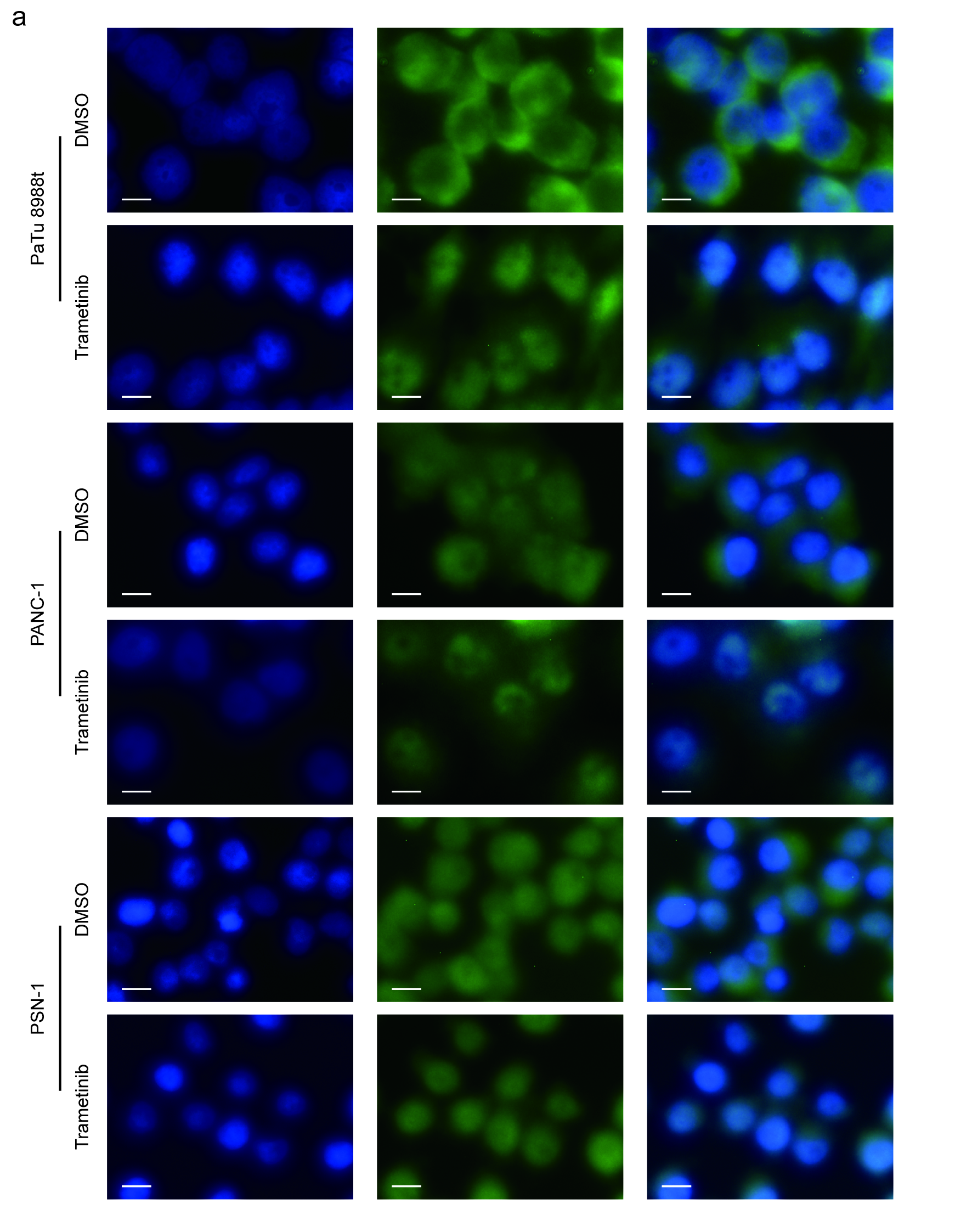

Supplement: Supplementary file 11 — supplementary Fig 3 [file 41420_2020_246_MOESM11_ESM.tif]

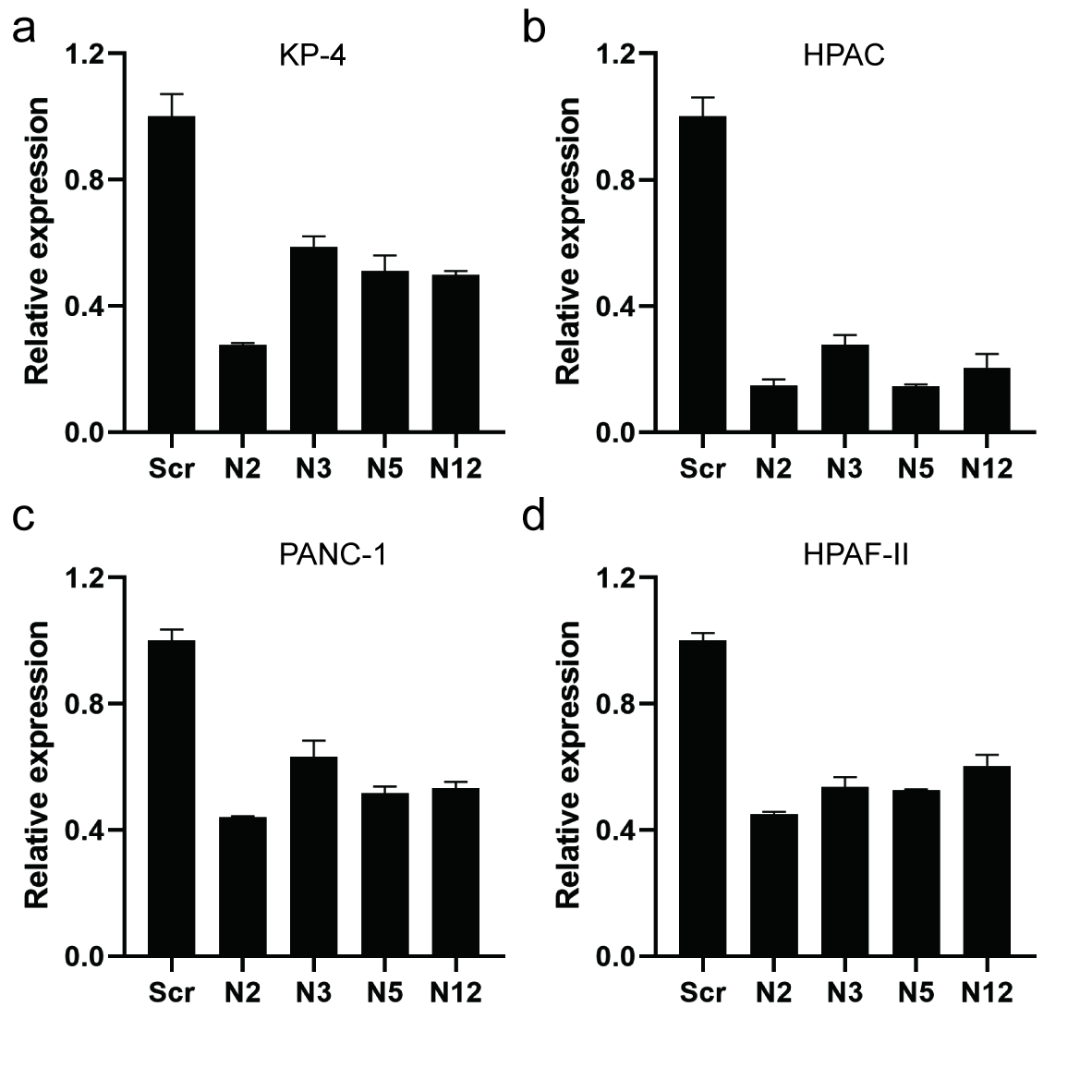

Supplement: Supplementary file 12 — supplementary Fig 4 [file 41420_2020_246_MOESM12_ESM.tif]

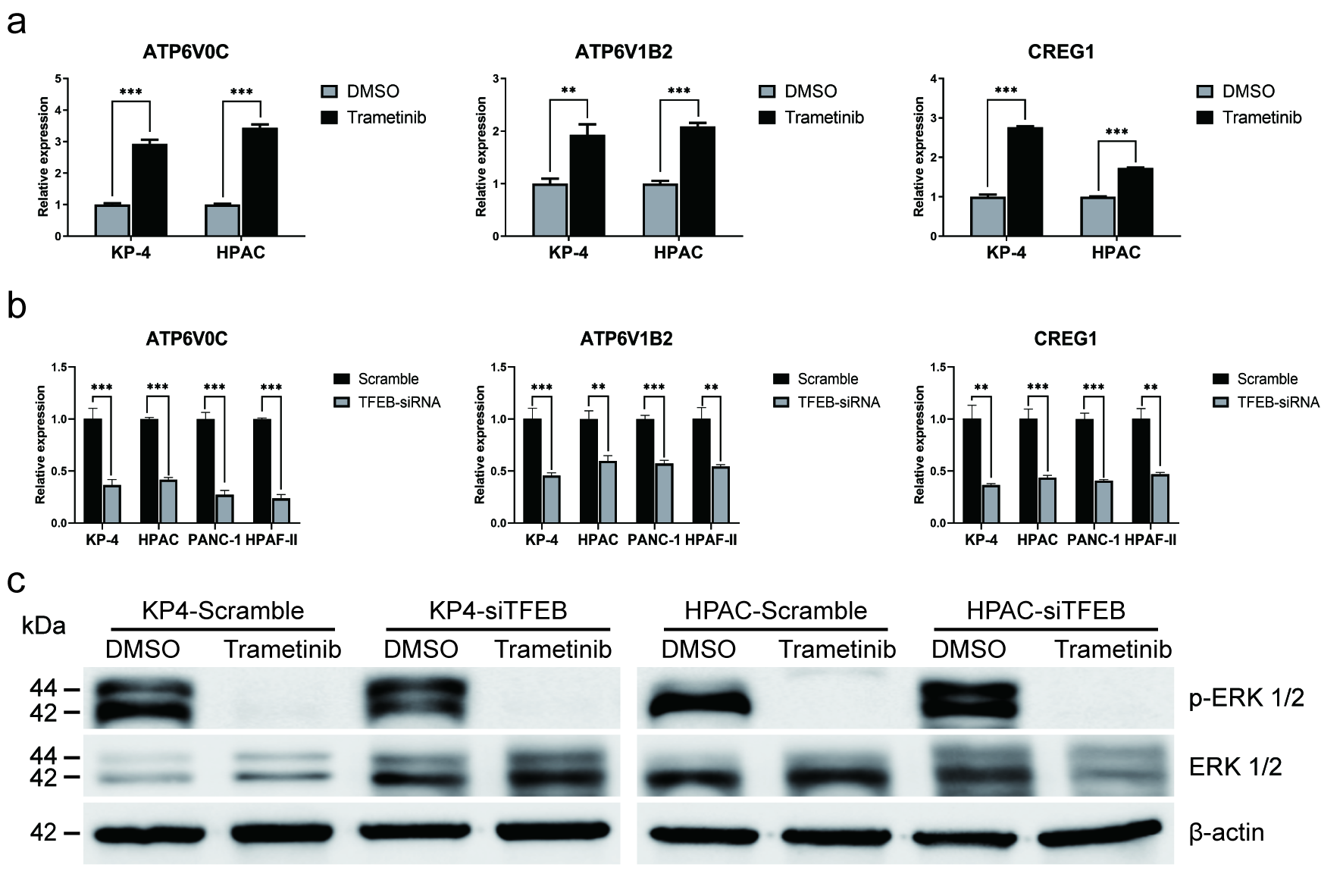

Supplement: Supplementary file 13 — supplementary Fig 5 [file 41420_2020_246_MOESM13_ESM.tif]

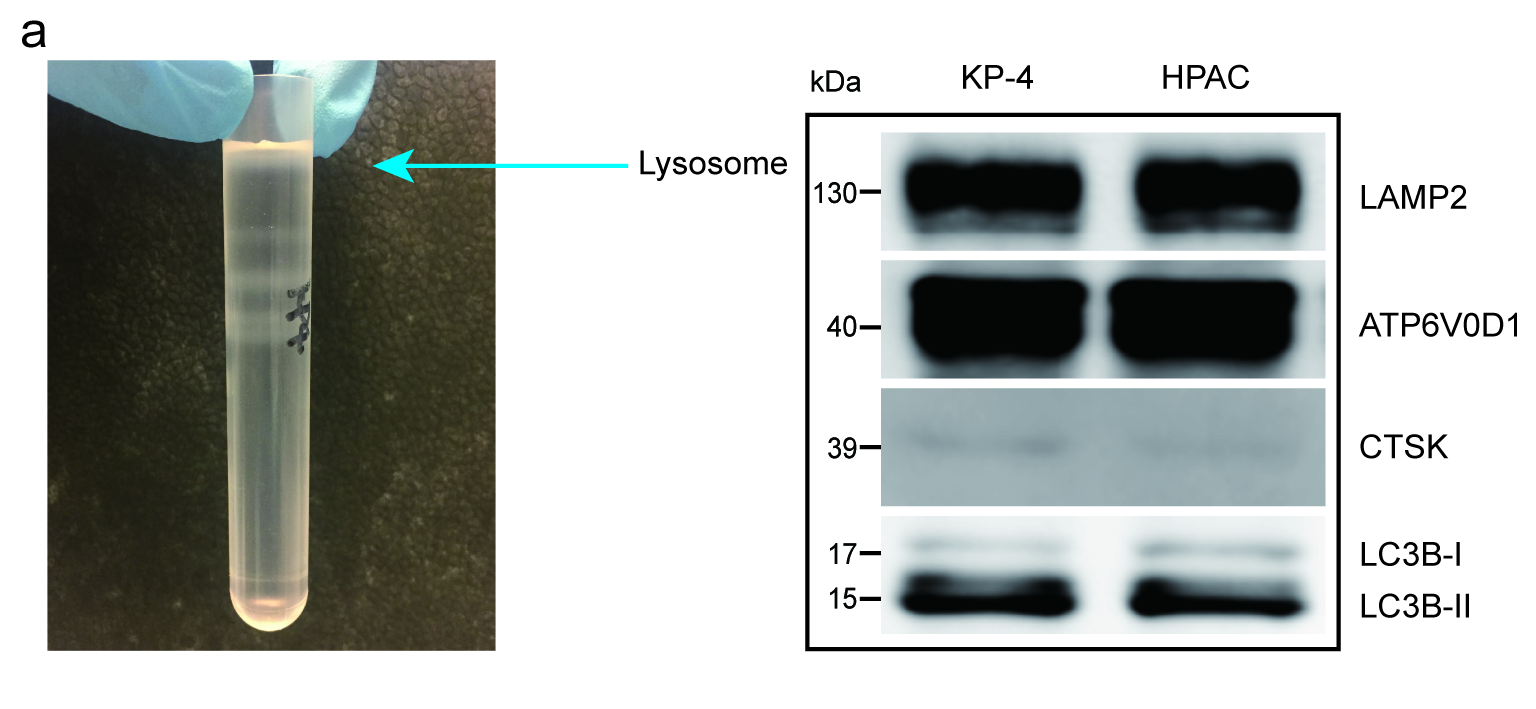

Supplement: Supplementary file 14 — supplementary Fig 6 [file 41420_2020_246_MOESM14_ESM.tif]

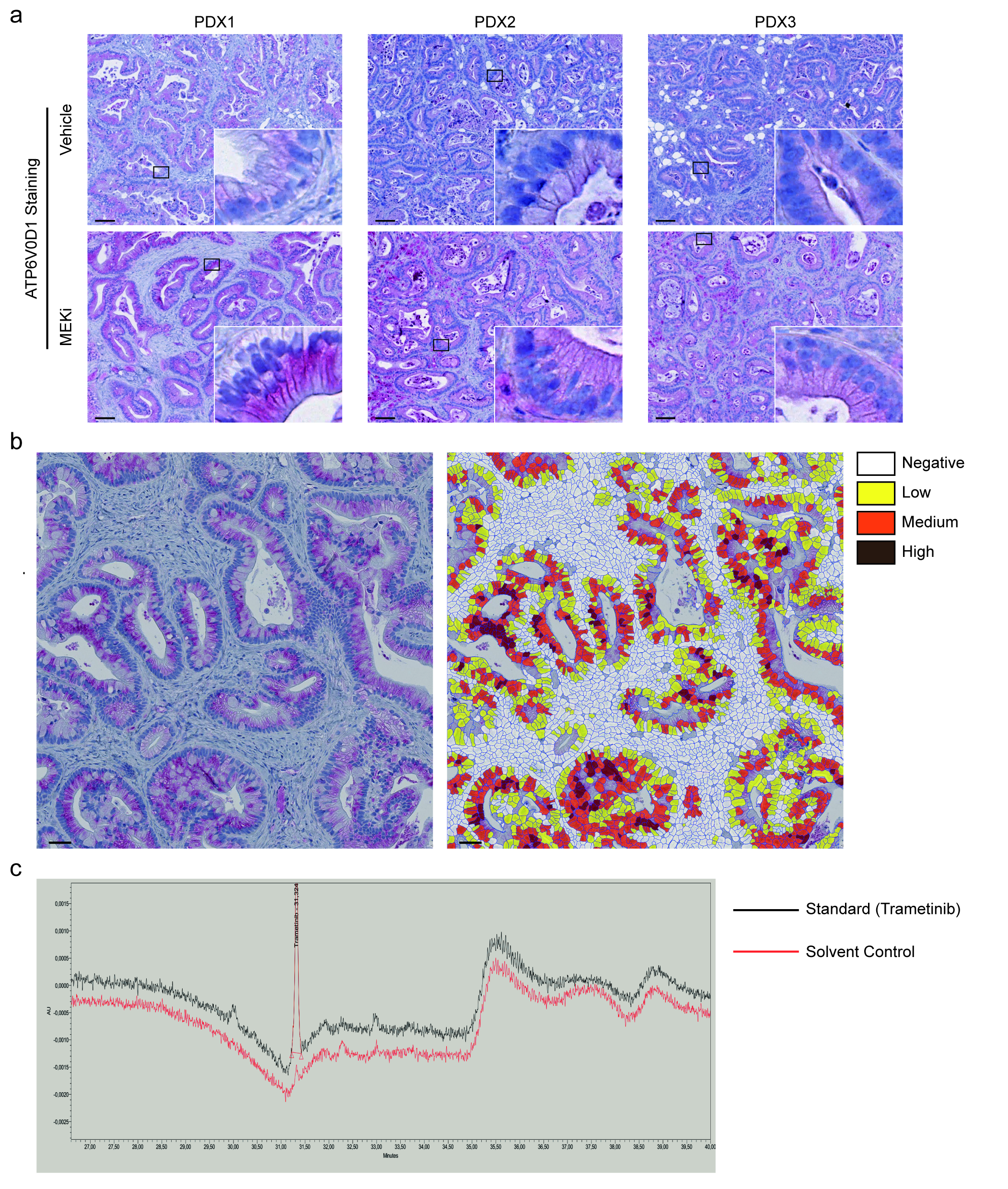

Supplement: Supplementary file 15 — supplementary Fig 7 [file 41420_2020_246_MOESM15_ESM.tif]
