# Supplementary material for: TFEB-mediated lysosomal biogenesis and lysosomal drug sequestration confer resistance to MEK inhibition in pancreatic cancer
Source: Cell Death Discov. 2020 Mar 11;6:12. doi: 10.1038/s41420-020-0246-7 (PMC7066197; doi:10.1038/s41420-020-0246-7)
Supplement: Supplementary file 16 — Supplementary figures and tables [file 41420_2020_246_MOESM16_ESM.docx]

**Supplementary information**

**TFEB-mediated lysosomal biogenesis and lysosomal drug sequestration confer resistance to MEK inhibition in pancreatic cancer**

Short title: Lysosomal drug sequestration mediates resistance to MEK inhibition

Ben Zhao^1,2^, Laura Dierichs^1,2^, Jiang-Ning Gu^1,2,3^, Marija Trajkovic-Arsic^1,2^, Ralf-Axel Hilger^4^, Konstantinos Savvatakis^1,2^, Silvia Vega Rubin de Celis^5^, Sven-Thorsten Liffers^1,2^, Samuel Peña-Llopis^1,2,6^, Diana Behrens^7^, Jens T. Siveke^1,2^* and Smiths Lueong^1,2^*

Page 2-8: Supplementary figure 1-7

9-10 Supplementary tables 1-4


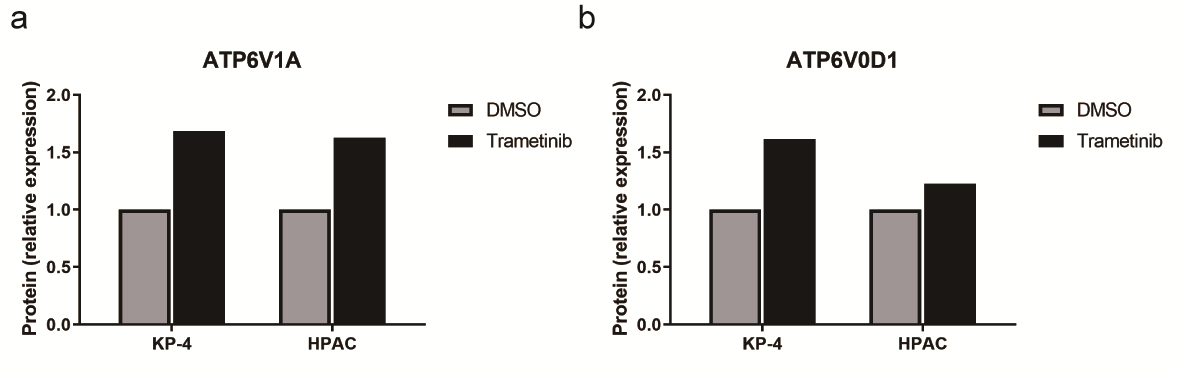


**Supplementary Figure1. Protein blot quantification for figure 3d**


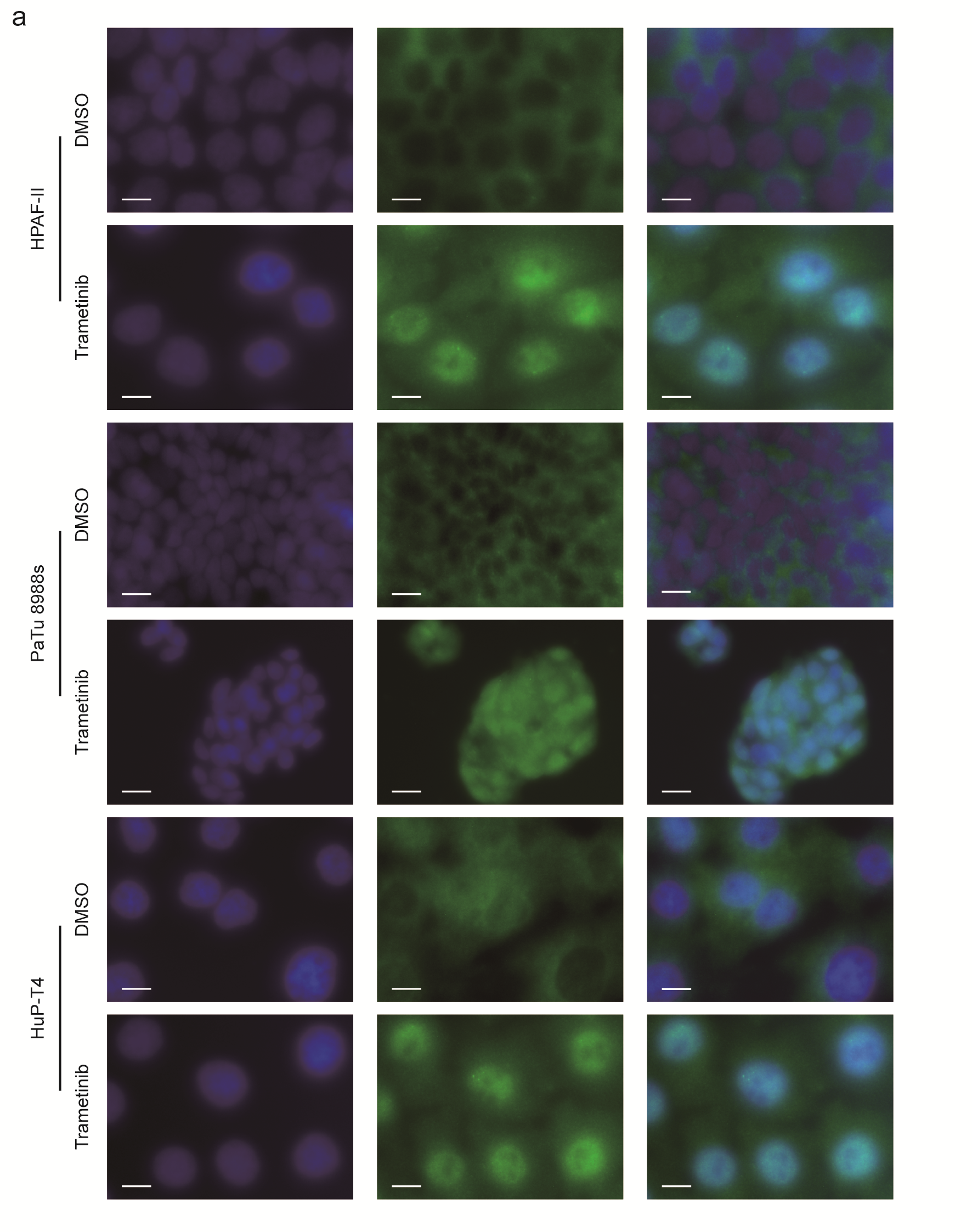


**Supplementary Figure 2. Immunofluoresence staining of TFEB in epithelial human PDAC cell lines with and without exposure to trametinib.** Cells were treated with single dose IC50 concentrations of trametinib for 72 hours and stained for TFEB


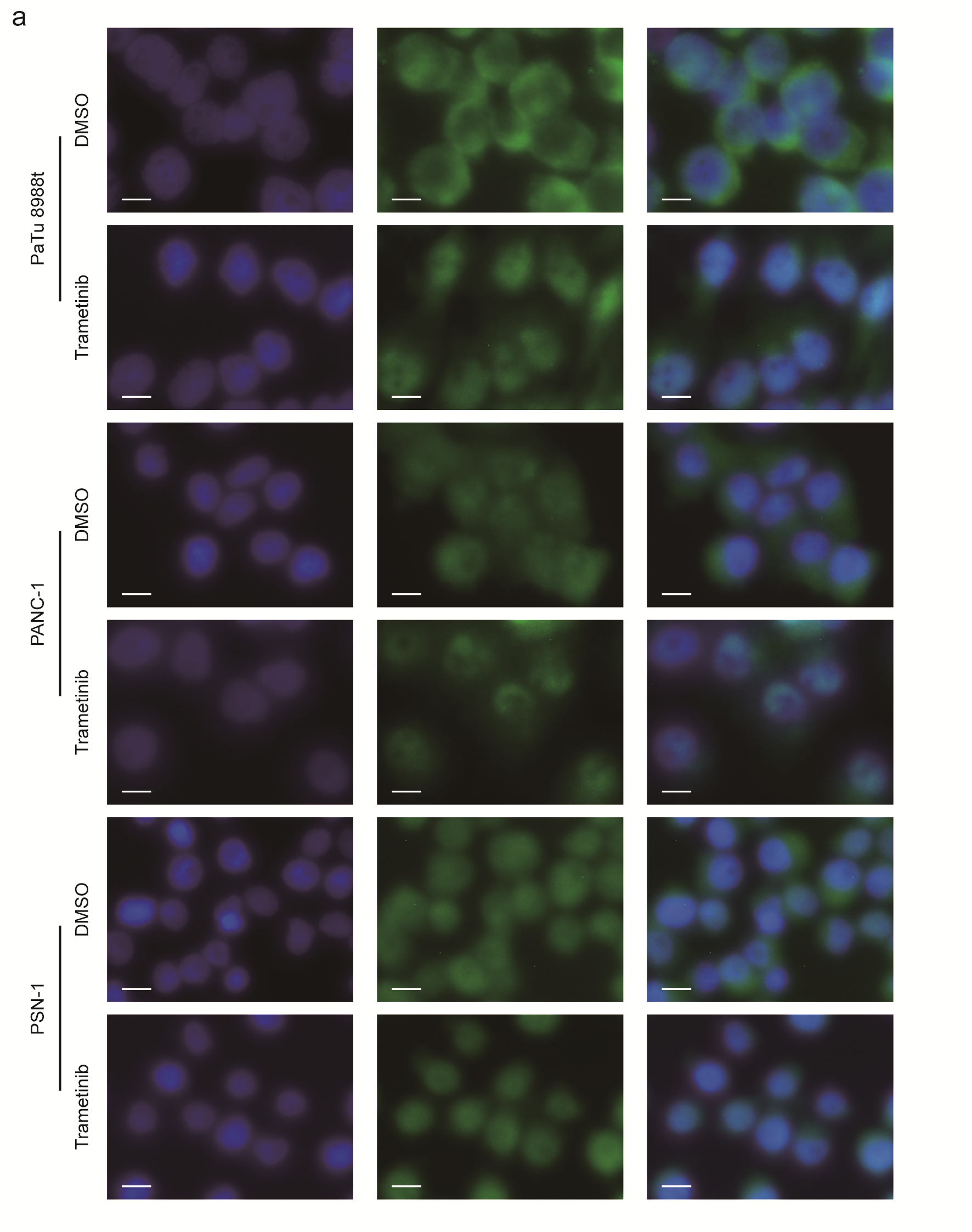


**Supplementary Figure 3. Immunofluoresence staining of TFEB in mesenchymal human PDAC cell lines with and without exposure to trametinib.** Cells were treated with single dose IC50 concentrations of trametinib for 72 hours and stained for TFEB

Supplementary Figure 5


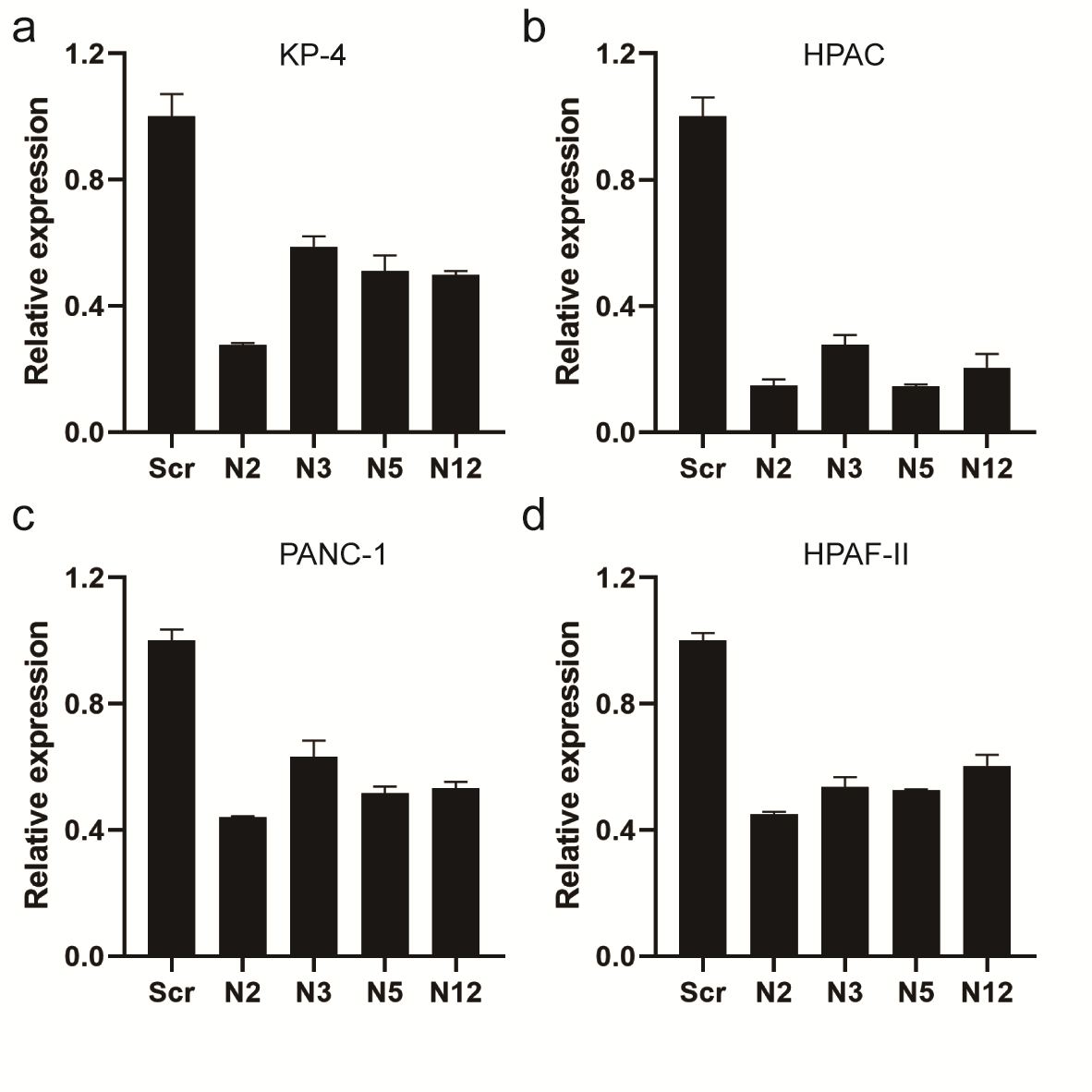


**Supplementary Figure 4. Identification of best-performing siRNA targeting the TFEB locus.** Transcript expression (qPCR) was performed for all supplied constructs in 4 individual human PDAC cell lines in two independent experiments.


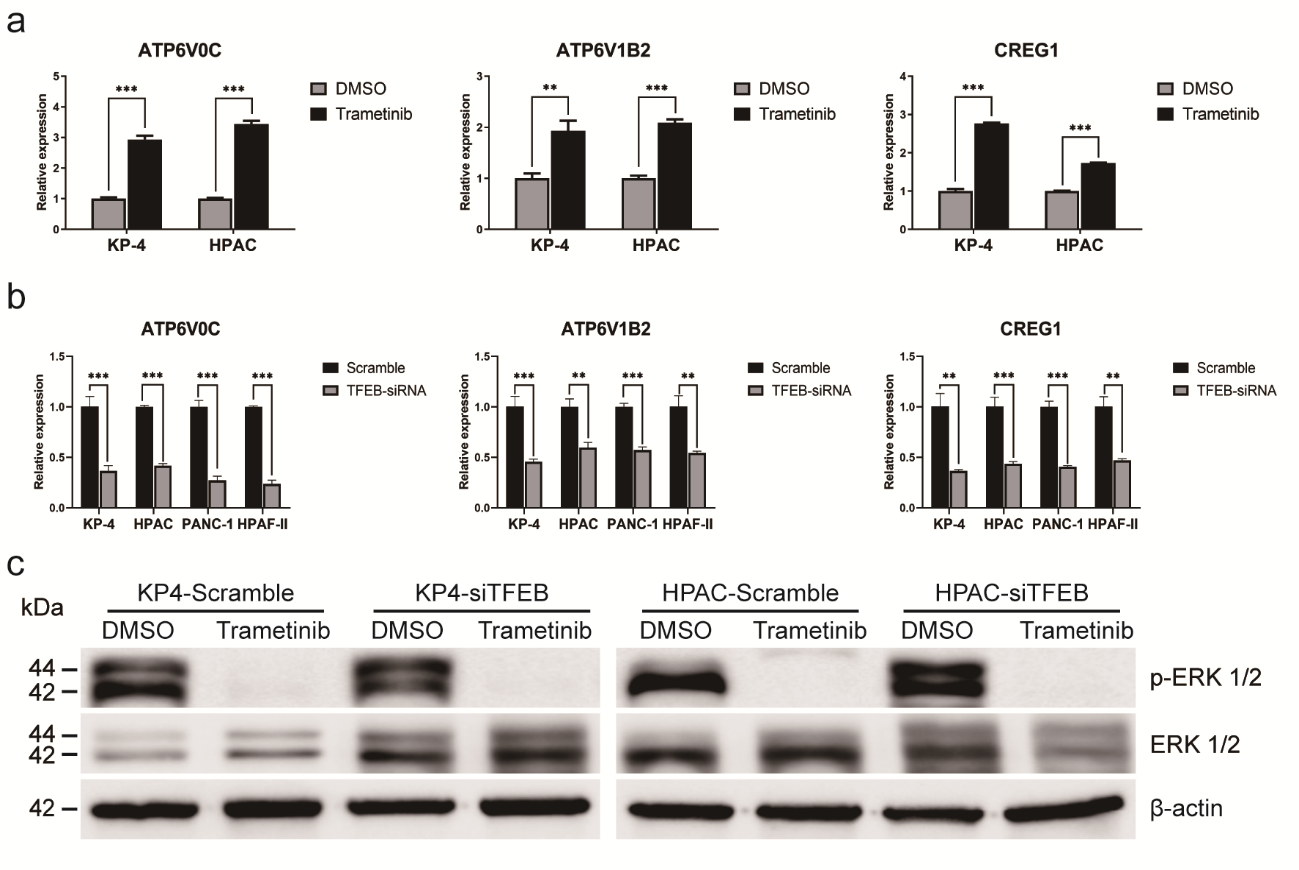


**Supplementary Figure 5.** **Gene and protein expression of direct TFEB targets. a)** transcript expression (qPCR, 3 independent experiments) of direct TFEB targets upon MEK inhibition. **b**) transcript expression (qPCR, 3 independent experiments) of direct TFEB targets upon TFEB knock down. **c**) protein blot illustrating downregulation of ERK1/2 phosphorylation upon MEK inhibition with trametinib. The ß-actin loading control has been replicated with figure 4e since those are the same lysates


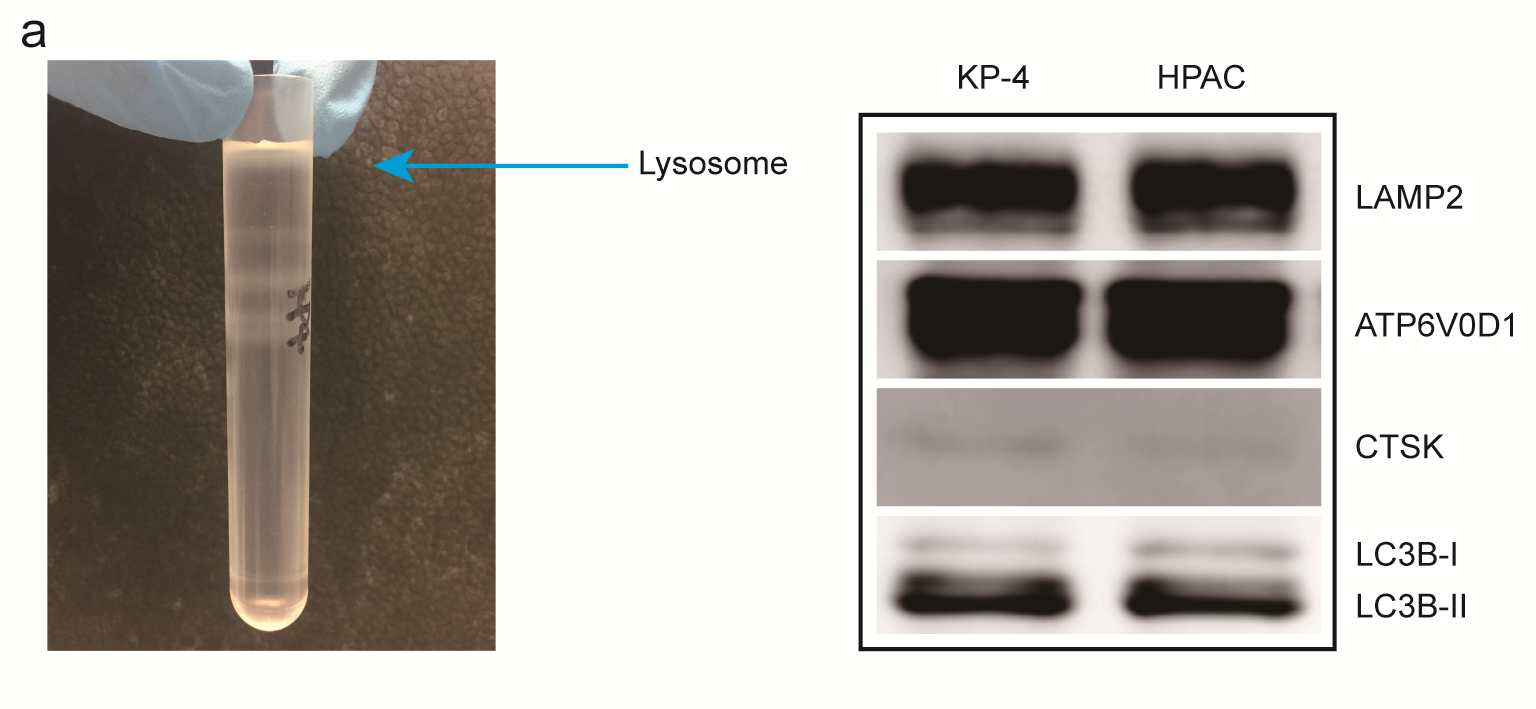


**Supplementary Figure 6. Isolation and characterizationof lysosomes.** An untracentrifuge tube showing a density gradient used for the isolation of lysosomes and the expected lysosome-containing band (left panel) and a protein blot for the lysosomal marker LAMP2 and ATPase ATP6V0D1 in the Isolated lysosome band (right panel)


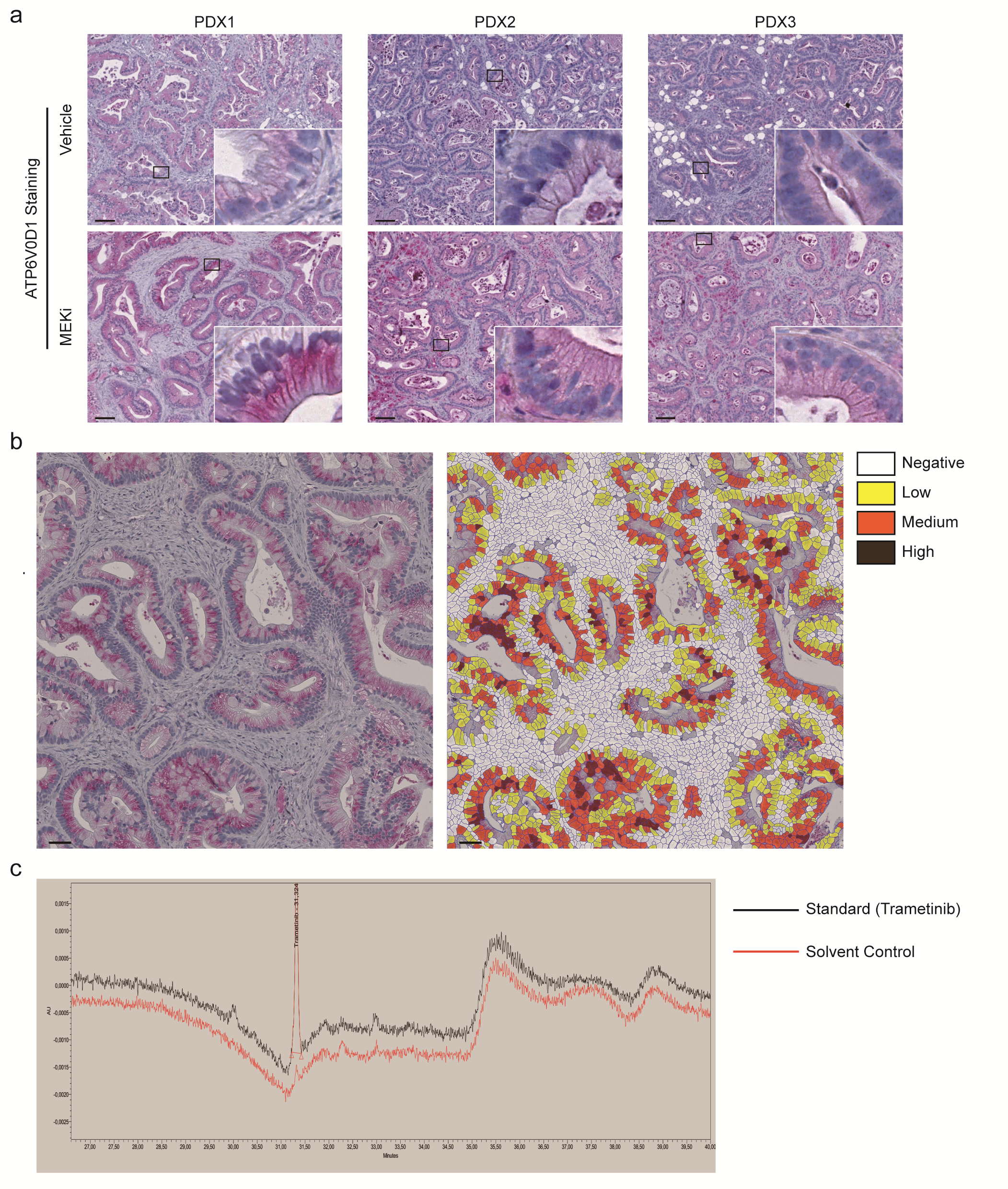


**Supplementary Figure 7.Immunostaining and signal intensity quantification. a)** immunohistochemical staining for the lysosomal ATPase ATP6V0D1 in human PDAC PDX tissues treat with the MEK inhibitor trametinib. b) representative original and software-transformed immunostaning image for LAMP1 in trametinib-treated human PDAC PDX. C) Mass spectrometry calibration standard used for the detection of trametinib in lysosomes.

**Supplementary table 1: List of siRNA used**

**Supplementary table 2: List of antibodies used**

| Antibody | Cat N° | Company | Host |
| --- | --- | --- | --- |
| LAMP1 | ab24170 | Abcam | Rabbit |
| LAMP2 | 49067 | Cell Signaling Technlogies | Rabbit |
| ATP6V0D1 | ab202899 | Abcam | Rabbit |
| ATP6V1A | ab199326 | Abcam | Rabbit |
| CTSD | 2284 | Cell Signaling Technlogies | Rabbit |
| TFEB | ab2636 | Abcam | Rabbit |
| TFEB | 4240 | Cell Signaling Technlogies | Rabbit |
| p44/42 | 4695 | Cell Signaling Technlogies | Rabbit |
| Phospho-p44/42 | 4376 | Cell Signaling Technlogies | Rabbit |
| ß-actin | ab8227 | Abcam | Rabbit |
| LC3II | ab51520 | Abcam | Rabbit |
| CTSK | ab19027 | Abcam | Rabbit |

| Target Gene | Cat N° | Company |  |
| --- | --- | --- | --- |
| Scramble | 1027310 | Qiagen |  |
| TFEB | SI00094969 | Qiagen |  |
| TFEB | SI00094976 | Qiagen |  |
| TFEB | SI03088568 | Qiagen |  |
| TFEB | SI05349267 | Qiagen |  |

**Supplementary table 3: Cell numbers used for screening**

| **Cell Line** | **Optimal numbers** |
| --- | --- |
| KP-4 | 3000 |
| HPAC | 3000 |
| HPAF-II | 4000 |

**Supplementary table 4: List of primers used**

| **Primer Name** | **Sequence 5'-->3'** |
| --- | --- |
| *ATP6V0C* Fwd | GTATGCTTCGTTTTTCGCCG |
| *ATP6V0C* Rev | CGATGATGCCAGCCATGACCAC |
| *ATP6V1A* Fwd | GAAACTTCTGGTGTGTCTGT |
| *ATP6V1A* Rev | CCATAATGCCAGGACCAAG |
| *CREG1* Fwd | CAAAAATCGTGACACCAGAAG |
| *CREG1* Rev | CTAAATTCACCACAGTCTGCTTC |
| *ATP6V1B2 Fwd* | GATGTGCAAGCCATGAAAGC |
| *ATP6V1B2* Rev | GCCAGCCAATGTCCAAAGTC |
| *LAMP1 Fwd* | TGCTGACGACAATGCATGAG |
| *LAMP1* Rev | CTCTCCGCACCGTACCC |
| *Vim Fwd* | TGAAGGAGGAAATGGCTCGTC |
| *Vim* Rev | GTTTGGAAGAGGCAGAGAAATCC |
| *CDH1 Fwd* | CGCATTGCCACATACACTCT |
| *CDH1* Rev | TTGGCTGAGGATGGTGTAAG |
| *CTSD Fwd* | CGGATGGACGTGAACTTGT |
| *CTSD* Rev | AGCCCTCCAGCCTTCTG |
| *ATP6V0D1* Fwd | CTCGTTGGCCAGGAAGTTAC |
| *ATP6V0D1* Rev | CCGACTACCTCAACCTGGTG |
