# Supplementary material for: TFEB-mediated lysosomal biogenesis and lysosomal drug sequestration confer resistance to MEK inhibition in pancreatic cancer
Source: Cell Death Discov. 2020 Mar 11;6:12. doi: 10.1038/s41420-020-0246-7 (PMC7066197; doi:10.1038/s41420-020-0246-7)
Supplement: Supplementary file 17 — Author contribution [file 41420_2020_246_MOESM17_ESM.pdf]

DECLARATION OF CONTRIBUTIONS TO ARTICLE

ADMC

Manuscript Number:	Journal Name:	
CDDIS-19-2952	*Cell Death & Disease*	(the 'Journal')

Proposed Title of the Contribution:

TFEB-mediated lysosomal biogenesis and lysosomal drug sequestration confer resistance to MEK inhibition in pancreatic cancer (the 'Contribution)

Author(s):

Ben Zhao, Laura Dierichs, Jiang-Ning Gu, Marija Trajkovic-Arsic, Ralf Axel Hilger, Konstantinos Savvatakis, Silvia Vega-Rubin-de-Celis, Sven-Thorsten Liffers, Samuel Peña-Llopis, Diana Behrens, Stephan Hahn Jens T. Siveke and Smiths S. Lueong (the 'Authors')

For all *CDDis* articles, each person named as an author in the published version must be able to show he or she has contributed substantially to the article.

Authorship credit should be based on 1) substantial contributions to conception and design, acquisition of data, or analysis and interpretation of data; 2) drafting the article or revising it critically for important intellectual content; and 3) final approval of the version to be published. Authors should meet conditions 1, 2 and 3.

Any person who cannot be shown to have made a substantial contribution to the article cannot be listed as an author in the final version. The name of any person who is deemed to have made a minor contribution can, however, appear in the Acknowledgments section of the article.

Please complete the table below to indicate the contributions of all named authors to the manuscript.

Author Full Name:	Specification of Contribution to the Manuscript:
Ben Zhao	Performed cell culture experiments on human PDAC cell lines and prepared figures
Laura Dierichs	Generated trametinib-resistant mouse PDAC cell lines and gene expression data
Jiang-Ning Gu	Performed short-term treatment of mouse PDAC cell lines
Marija Trajkovic-Arsic	Generated refamatinib-treated mouse tissue samples
Ralf Axel Hilger	Performed mass spectrometry
Konstantinos Savvatakis	Processed all tissue Section
Silvia Vega-Rubin-de-Celis	Provided reagents and proof-read the manuscript
Sven-Thorsten Liffers	Organized tissue samples from Human PDAC xenografts
Samuel Peña-Llopis	Provided reagents and proof-read the manuscript
Diana Behrens	Generated human PDAC Xenografts and treatement
Stephan Hahn	Provided human primary PDAC cell lines
Jens T. Siveke	Provided funding for the experiments
Smiths S. Lueong	Designed the study, did experiments, analyzed data and wrote the manuscript

Please complete the table below to indicate the contributions of all named authors to the figures.

Figure 1:

Laura Dierichs
Jiangnig Gu
Marija Trajkovic-arsic
Smiths Lueong
Konstantinos Savvatakis

Figure 2:

Ben Zhao

Figure 3:

Ben Zhao
Smiths Lueong

Figure 4:

Ben Zhao
Smiths Lueong

Figure 5:

Ben Zhao
Smiths Lueong
Ralf Axel Hilger

Figure 6:

Ben Zhao
Smiths Lueong
Stephan Hahn
Diana Behrens
Konstantinos Savvatakis
Sven-Liffers

Signed for and on behalf of the Author(s):	Print Name:	Date:
[signature]	Smiths S. Lueong	Essen the 20th August 2019
